# Modelling local gene networks increases power to detect *trans*-acting genetic effects on gene expression

**DOI:** 10.1186/s13059-016-0895-2

**Published:** 2016-02-24

**Authors:** Barbara Rakitsch, Oliver Stegle

**Affiliations:** European Molecular Biology Laboratory, European Bioinformatics Institute, Wellcome Trust Genome Campus, Hinxton, Cambridge, UK

**Keywords:** eQTL mapping, Causal reasoning, Genome-wide association studies, Confounder correction, Competing exposures

## Abstract

Expression quantitative trait loci (eQTL) mapping is a widely used tool to study the genetics of gene expression. Confounding factors and the burden of multiple testing limit the ability to map distal *trans* eQTLs, which is important to understand downstream genetic effects on genes and pathways. We propose a two-stage linear mixed model that first learns local directed gene-regulatory networks to then condition on the expression levels of selected genes. We show that this covariate selection approach controls for confounding factors and regulatory context, thereby increasing eQTL detection power and improving the consistency between studies. GNet-LMM is available at: https://github.com/PMBio/GNetLMM.

**Electronic supplementary material:**

The online version of this article (doi:10.1186/s13059-016-0895-2) contains supplementary material, which is available to authorized users.

## Background

Expression quantitative trait loci (eQTL) mapping is an approach to study the genetic component of transcriptomic variation between individuals. By correlating genetic variants with gene expression profiles of individual genes, it has been possible to establish genome-wide maps of genetic effects on gene expression, both in model systems [1–3] and in human [4–9]. Several statistical methods have been proposed to maximize the power to detect *cis*-acting eQTLs, which are proximal to the regulated genes and typically have large effects [10–12]. In contrast, the robust identification of distal *trans* genetic effects (e.g. [13–16]) remains a major challenge. This is because *trans* eQTLs tend to have smaller effect size, are frequently tissue- and context-specific [17, 18], and due to genome-wide tests impose a severe multiple-testing burden. Despite these limitations to map *trans* eQTLs, heritability estimates suggest that collectively *trans* genetic effects explain a substantial proportion of the overall gene expression variance [15, 19], which cannot be explained by *cis* eQTLs. Moreover, if reliably detected, *trans* eQTLs have the potential to deliver new insights into (downstream) genetic regulation, for example by identifying genetic effects that are mediated via *cis*-regulated genes [20, 21]. Finally, by overlaying distal eQTLs with disease risk loci identified through genome-wide association studies (GWAS), *trans* eQTL maps will be an invaluable resource to identify therapeutic targets for human diseases [22, 23].

One principle to improve power to map eQTLs is to account for competing exposures, which are sources of variation other than the genetic variant being tested. In particular non-genetic covariates, if not accounted for, can mask genetic signals, which impacts eQTL discovery. Recently, methods based on factors analysis and related latent variables models [24] have been proposed to reconstruct a typically small number of unobserved (confounding) factors from the expression data itself. These inferred latent variables can then be accounted for in genetic models, either as fixed effect covariates [11, 25] or as random effects within the linear mixed model (LMM) framework [10, 12, 26]. These approaches have been successfully applied to reduce the effect of unmeasured environmental factors or batch, such that genuine genetic signal can stand out to a greater extent. Importantly, however, while these methods are widely used for *cis* eQTL mapping [7, 9–12], there are major pitfalls when modelling unobserved covariates in the analysis of *trans* effects. In particular, there is a risk that models such as factor analysis falsely capture genuine genetic signals from regulatory hotspots that affect larger sets of genes. In such instances, it has been shown that the inferred latent variables are heritable themselves and can be mapped as quantitative traits [27]. Because of this challenge, existing methods to correct for confounding are difficult to apply to the analysis of *trans* eQTLs [12, 28, 29].

Related concerns have also been reported in the context of physiological phenotypes [30, 31]. There are fundamental challenges when conditioning on heritable covariates as cofactors, as this can lead to reduced statistical power or introduce spurious associations [32, 33]. This effect is well documented in the context of binary [34] and quantitative traits [33], and also in the genetic analysis of molecular phenotypes [28].

To address this, we here propose gene network LMM (GNet-LMM), a network guided approach to account for hidden variation in *trans* eQTL analyses. GNet-LMM identifies directed relationships in local gene regulatory networks to select covariates for eQTL association tests. Importantly and unlike previous methods to explain hidden variation in eQTL studies, GNet-LMM does not suffer from the risk of falsely explaining away genetic signals. The gene selection in GNet-LMM borrows principles from causal reasoning to identify a subset of genes that tag confounding or regulatory context, which are selected for every SNP–gene association test. The resulting adjustment is distinct from feature selection methods that have been proposed in GWAS [35], where the causal structure is known a priori (phenotypes cannot directly alter genotype) and hence correlation-based selection criteria are sufficient. Our approach is also related to methods that use causal tests, either to identify mediating genes [20] or to infer molecular networks downstream of eQTLs [36]. Whereas previous applications of network reconstruction have mainly been focused on obtaining mechanistic interpretations, here we show that local causal network reconstruction can help to improve power for detecting genetic associations.

We first validate the model on synthetic data before applying it to real data from mouse and human eQTL studies. Consistently across these applications, we find that GNet-LMM provides increased statistical power, yields associations that are enriched for known pathways and enhances the replicability of *trans* eQTLs between studies.

## Results and discussion

**Fig. 1:**
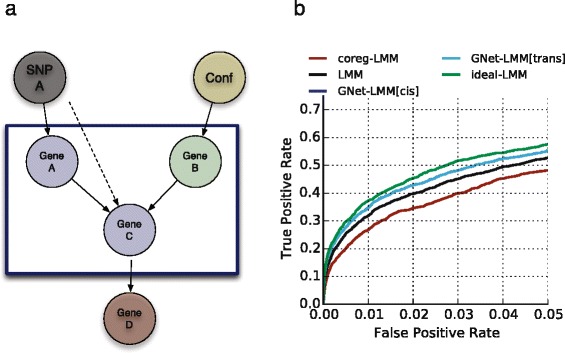
GNet-LMM model illustration and basic simulation experiment. **a** Graphical model representation of the GNet-LMM algorithm. For each SNP A–gene C *trans* association test, GNet-LMM identifies and conditions on exogenous genes with incoming edges (*green*, *Gene B*) but not on genes with outgoing edges (*red*, *Gene D*). Exogenous genes either tag confounding sources of variation (*Conf*) or regulatory effects between genes. To define exogenous genes, GNet-LMM tests for V-structures gene A - > gene C < - gene B (*blue box*) that are linked to SNP A via gene A. **b** True positive versus false positive rate when considering alternative methods applied to 1000 synthetic eQTL datasets that were simulated assuming a regulatory structure as in (**a**). Shown are results obtained from standard LMM without conditioning (*LMM*), an LMM that exclusively conditions on true exogenous genes (gene B, *Ideal-LMM*), an LMM that conditions on co-regulated genes (gene D, *coreg-LMM*) and the GNet-LMM algorithm that uses the V-structure approach to determine the set of exogenous genes for conditioning

It is well established that accounting for covariates and confounding factors can help to increase power in eQTL analyses [11, 25]. The majority of such factors are not directly observed, so one is limited to condition on proxies, such as the expression levels of individual genes or latent variables estimated from expression data. Importantly, correlation-based criteria to select covariates to include in genetics models are not sufficient. For example, Fig. 1b shows the power to detect true SNP A–gene C *trans* associations when including genes with different causal relationships to the *focal* gene C as covariates. The association signal is increased when conditioning on genes with incoming edges (gene B), but markedly decreased when accounting for genes with outgoing edges (gene D), even though both genes were simulated to have a similar correlation with gene C.

If the true regulatory dependencies were known a priori, an optimal strategy to select covariates could be straightforwardly defined based on the directed edge information. Our objective is to condition on all genes B that (i) have a causal effect on the *focal* gene C *and* (ii) are independent of the genetic variant SNP A (Fig. 1a). In the following we term genes that satisfy these two conditions *exogenous factors*. These factors explain competing exposures, which can either be confounding factors or gene regulatory effects. Importantly, the true gene–gene network is unknown in practice. Consequently, we need to identify exogenous genes from the observed data.

### GNet-LMM enables selective conditioning on exogenous genes

GNet-LMM first fits local directed gene regulatory networks to then identify exogenous genes. Briefly, for each (SNP A, gene C) pair, the algorithm initially tests for quartets (SNP A, gene A, gene C, gene B) whose regulatory dependencies can be described by the directed acyclic graph as shown in Fig. 1a. To do so, we test for gene triplets that form a so-called V-structure (gene A - > gene C < - gene B), which are defined by two *directed* regulatory edges to the focal gene C, one from gene A and one from gene B (Fig. 1a). A set of basic statistical dependence criteria can be used to identify these structures, requiring dependence between both A–C and B–C and independence between genes A and B, which become dependent after conditioning on the gene C (“Materials and methods” and [37]). The key outcome of the V-structure test is to define a set of direct regulatory relationship from which *exogenous* genes can be identified. For the sake of computational efficiency and to increase robustness, GNet-LMM does not consider all possible pairs of genes A and B (which would scale quadratically in the number of genes for each eQTL test). Instead, we restrict gene A to genes with a significant *cis* or *trans* association to SNP A (see also [20], where a similar approach has been used in a different context). In the following, we denote these two search strategies GNet-LMM[cis] and GNet-LMM[trans]. Subsequently, to test for SNP A–gene C associations, the algorithm conditions on the set of genes B that satisfy the V-structure criterion for the given (SNP A, gene C) pair (“Materials and methods”).

To illustrate the benefits of selecting exogenous genes using GNet-LMM, we initially considered a basic simulation experiment to assess the power of alternative methods to detect true associations between a distal (*trans* acting) genetic variant (SNP A) and the expression level of a *focal* gene (gene C). We simulated genetic effects that are mediated by a *cis* association (SNP A–gene A), conferring the genetic effect via an indirect (*trans*) effect to gene C. Additionally, we also simulated effects due to unmeasured covariates, such as environmental factors or batch effects. When testing for SNP A–gene C *trans* associations, conditioning on true exogenous genes (gene B) increased power (Fig. 1b; LMM versus ideal-LMM), whereas conditioning on genes that are co-regulated by the same genetic variant (gene D) markedly decreased power (Fig. 1b; LMM versus coreg-LMM). Encouragingly, when using exogenous genes identified using local network inference based on V-structures (GNet-LMM), the power to detect eQTLs was similar to an ideal model that uses the simulated ground truth to define exogenous genes (Fig. 1b; Figure S1 in Additional file 1; GNet-LMM versus ideal-LMM).

If the association between SNP A and gene C is neither mediated by a *cis-* nor by a *trans-*gene, no V-structure is identified and the method reverts to a standard LMM (Figure S2 in Additional file 1). There is a concern that conditioning on covariates (genes) that are heritable themselves can lead to spurious associations, for example if gene B is itself regulated by SNP A [33]. These instances can be ruled out by additional independence tests, resulting in robust selection of exogenous genes (“Materials and methods”; Additional file 1: Figure S3). We also assessed the effect of spurious correlations between groups of genes due to (unobserved) confounders, again observing that the model remained statistically calibrated (“Materials and methods”; Figure S3 in Additional file 1).

### Simulation study

Next, we considered genotypes from the 1000 Genomes project [38] and generated simulated expression profiles that mimic regulatory dependencies from real eQTL studies. Using genotype data from 379 individuals of European ancestry (CEU, FIN, GBR, IBS, TSI), we generated gene expression levels assuming a combination of *cis* and *trans* genetic effects as well as effects due to external confounding factors (“Materials and methods”). We varied the structure of the regulatory network, as well as the type and the magnitude of the confounding effects. We compared GNet-LMM to a standard LMM and established latent variable models to account for confounding in eQTL analyses, including two approaches based on principal component analysis (PC-LMM, PCselect-LMM [13, 39]), and random effect models (ICE-LMM [10]). We again considered an ideal model, where the simulated network topology was used to condition on the true set of *exogenous* genes (ideal-LMM) and where all confounding factors were included in the model. All methods were assessed in terms of their power to detect *trans* eQTLs (at false positive rate < 5 %).

**Fig. 2:**
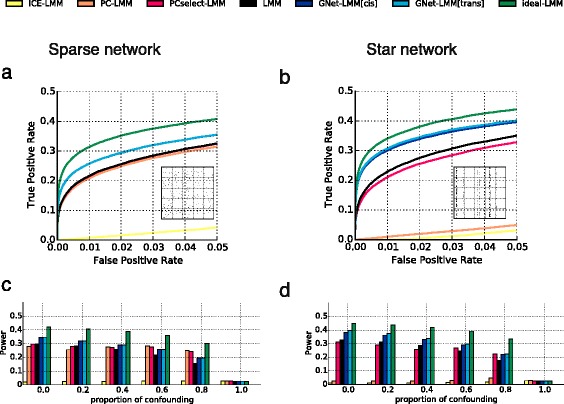
Benchmark of alternative eQTL association methods on simulated datasets. **a**, **b** Receiver operating characteristic (ROC) for alternative methods to detect eQTLs in sparse (**a**) and star-shaped simulated regulatory networks (**b**), assuming no confounding factors. **c**, **d** Power comparison when increasing the relative effect of confounding, either assuming sparse (**c**) or star-shaped network topologies (**d**). Compared are a standard LMM without conditioning (*LMM*), an LMM that exclusively conditions on true exogenous genes (*Ideal-LMM*), adjustment based on principal components (*PC-LMM*), adjustment based on principal components with selection (*PCselect-LMM*) and GNet-LMM. Power is defined as the area under the ROC curve for a false positive rate below 5 %

Initially, we examined the sensitivity of the considered methods with respect to the architectures of regulatory networks without confounding, either simulating a sparse, unstructured network between the genes (Fig. 2a) or considering a star-shaped network with regulatory hubs (Fig. 2b) (“Materials and methods”). In both settings, GNet-LMM considerably improved power compared with a standard LMM (GNet-LMM[cis/trans] versus LMM) (Fig. 2a, b). In contrast, we observed that methods based on principal component analysis (PCA) had either no benefit compared with a standard LMM (Fig. 2a) or even reduced power in star-shaped network topologies (Fig. 2b). This reduction in power is because covariates inferred using PCA tend in part to capture genetic signals, thereby explaining away the effect of genetic master regulators with widespread downstream effects (see also discussion in [10, 12]). This deficiency of a vanilla PCA approach was reduced when selecting principal components (PCs) that were not associated with genetic variants (Fig. 2b; PCselect-LMM). However, this approach was still inferior to a conventional LMM, and considerably less powered than GNet-LMM. This suggests that PC-based adjustment in general has little benefit if no confounding factors are present. Finally, ICE-LMM appeared to be the most conservative model, explaining away large proportions of the actual genetic signal. This behaviour has previously been noted, e.g. [12] and addressed in recent extensions [29].

Next, we investigated the performance of these methods when simulating increasingly strong confounding effects independent of the network (“Materials and methods”; Figure S4 in Additional file 1). While PCA-based methods tended to increase power for sparse regulator networks (Fig. 2c), both PC-LMM and PCselect-LMM were unable to disentangle correlated genetic effects due to master regulators and confounding in star-shaped network topologies (Fig. 2d). In contrast, GNet-LMM consistently improved power compared with the LMM baseline (Fig. 2c, d) and performed markedly better than PCA adjustment in a wide range of parameter regimes. We also observed that associations of PCs with genetic factors were frequently weak and difficult to detect (Figure S5 in Additional file 1). This is a likely explanation of why methods that select PCs based on association criteria are less reliable than V-structure selection in GNet-LMM.

We also considered additional experiments, altering the proportion of gene expression variance due to *trans* eQTLs, the overall variance explained by genetic and confounding factors, the average number of confounders that effect any one gene and the total number of simulated confounding factors. Across these scenarios, GNet-LMM consistently increased statistical power compared with other methods (Figure S6 in Additional file 1). One limitation of GNet-LMM is in the regime of tightly correlated gene expression levels, which can occur if one dominant factor, such as a large hidden batch effect, explains most of the gene expression variance. This correlation hampers the ability to identify V-structures and hence GNet-LMM reverts to a standard LMM.

### Application to eQTL datasets from mouse and human

**Fig. 3:**
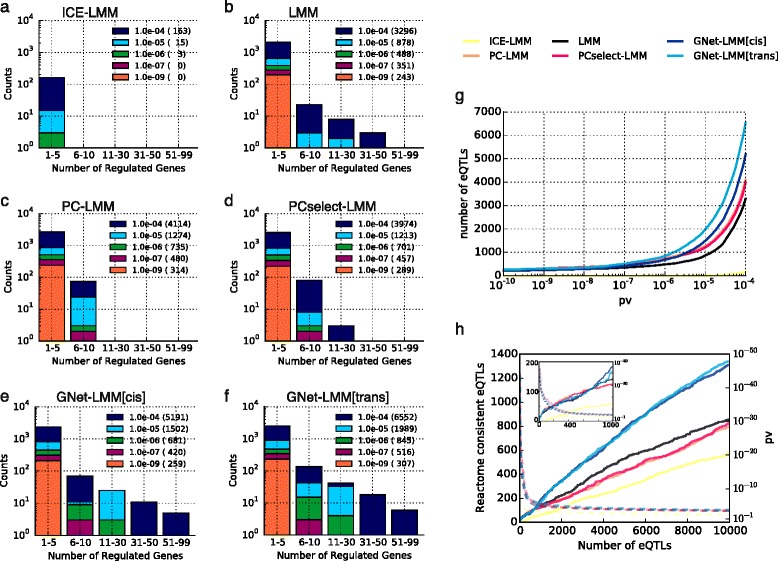
Application of alternative eQTL association methods to the mouse dataset. **a**–**f** The number of detected *trans* eQTLs stratified by the number of regulated genes (hotspot size). Results for alternative significance thresholds are colour coded. While PC-based approaches increased power to detect variants that regulate a small number of genes (**c**, **d**), GNet-LMM (**g**, **f**) maintained a profile of eQTL hotspots that was similar to the results from an unadjusted analysis (LMM) (**b**). **g** Total number of *trans* associations retrieved by alternative methods as a function of the significance threshold. **h** Gene enrichment analysis of *trans* eQTLs using Reactome pathways. *Solid lines* show the proportion of Reactome-consistent *trans* eQTLs as a function of the rank of individual associations sorted by their significance level (“Materials and methods”). *Dashed lines* denote the corresponding *p* value threshold. eQTLs identified by GNet-LMM were markedly more enriched for Reactome pathways than eQTLs retrieved by alternative methods

Next, we revisited an eQTL dataset of hippocampus gene expression profiled in a panel of 467 heterogeneous stock mice [40] (expression levels for 3740 genes/12,545 genetic markers after quality control; “Materials and methods”). All considered methods except for ICE-LMM were well calibrated (Figure S7 in Additional file 1), again attesting that the ICE model is conservative. Because of the large haplotype blocks in this population, we classified associations as *trans* eQTLs if the distance from the transcription start site of the gene exceeded 20 Mb. In line with the results obtained on simulated data, we observed that accounting for PC-based covariates substantially reduced the power to identify regulatory hotspots (Fig. 3c, d), although the overall number of *trans* eQTLs increased compared with a standard LMM (Fig. 3g). These results were consistent with our simulation study, suggesting that, in general, PCs capture a combination of confounding factors and genetic signals from *trans* hotspots. As a result, we observed that adjustment using PCs led to a detection bias, where the power to detect associations in *trans*-hubs decreased whereas the power to identify non-structured (sparse) associations increased.

The fact that PCs explain heritable genetic signal was also apparent when considering PCs as quantitative traits and mapping their genetic effects. QTL mapping of the PCs themselves revealed suggestive associations (*p* < 1e–4) that coincided with the locations of the *trans* hotspots that were explained away by the PC-based adjustment (Figure S8 in Additional file 1). In contrast, GNet-LMM recovered all *trans* hotspots identified by a standard LMM and revealed additional hotspots (Fig. 3e, f; Figure S8 in Additional file 1).

To objectively assess the associations retrieved by GNet-LMM and alternative methods, we tested to what extent the discovered *trans* eQTLs were consistent with Reactome pathways [41]. Briefly, for each method we gauged individual SNP–gene associations by assessing whether the gene tagged by the eQTL variant (i.e. its *cis* gene) and the focus gene (regulated in *trans*) were annotated in at least one common pathway. Although such an approach may miss both true positive and false positive associations, we reasoned that the average consistency with known pathways is a suitable measure to compare eQTL-mapping approaches (see also [42, 43] where a similar strategy has previously been employed). Reassuringly, the *trans* eQTLs detected by GNet-LMM were substantially more enriched for known pathways (Fig. 3h) than those identified by alternative methods. Additionally, we repeated this enrichment test when stratifying individual loci based on the presence or absence of a *cis*-acting anchor gene. While globally, the enrichment of GNet-LMM[trans] was similar to GNet-LMM[cis], the method performed better in genomic regions without *cis-*mediating anchor genes, confirming that *trans* associations can be used as anchors to detect V-structures (Figure S9 in Additional file 1). Finally, we considered a permutation experiment to investigate the statistical calibration using an empirical null distribution for the final association test, but retaining the identical set of exogenous genes as identified in the full model. This analysis showed that the test statistics on data without signal were well calibrated, both for variants with and without exogenous genes (Figure S10 in Additional file 1), thereby confirming the calibration results on simulated data (Figure S3 in Additional file 1).

In summary, our results show that GNet-LMM is a robust and powerful approach to detect *trans* eQTLs. It is important to note that for the discovery of *cis* eQTLs, PCA-based approaches increased power to detect associations as previously reported, whereas GNet-LMM is by design identical to a standard LMM (Figure S11 in Additional file 1).

Finally, we applied GNet-LMM to the QTL dataset of the Cardiogenics Transcriptomic Study [44, 45]. We considered genotype markers from 376 healthy individuals and tested for genome-wide *trans* eQTLs affecting gene expression levels of 15,340 probes in monocytes. Again, all methods except for ICE were well calibrated (Figure S12 in Additional file 1). Overall, PC-based methods identified a larger number of *cis* associations than a standard LMM (Figure S13 in Additional file 1), and both PC-based methods and GNet-LMM increased power to detect *trans* effects (Figure S14 in Additional file 1).

Consistent with previously results obtained using the same dataset [45] and confirmed in an independent study [18], we identified a master regulator *LYZ* at the locus 12q15 (rs6581889). *LYZ* is known to be under strong genetic control by a *cis*-eQTL in monocytes and encodes the lysozyme enzyme. Lysozyme is important for immune defence and catalyses the cutting of polysaccharide chains of bacteria cell walls [46]. Downstream of the marker rs6581889, the standard LMM identified 127 *trans*-genes (*p* < 0.01, Bonferroni adjusted, accounting for the total number of tests). In line with the results obtained on the mouse dataset, substantially fewer *trans*-genes were found when using ICE-LMM (2), PC-LMM (117) or PCselect-LMM (116). In contrast, GNet-LMM[cis] and GNet-LMM[trans] both increased power to detect *trans* genes, identifying 214 and 218 genes in association with the LYZ locus, respectively (see Figure S15 in Additional file 1 for results using other significance thresholds). GNet-LMM recovered all eQTLs detected using a standard LMM and an analysis using the STRING database [47] suggested that the additional eQTL genes are embedded within functionally consistent networks (Figure S16 in Additional file 1).

As a complementary approach to validate eQTLs identified by GNet-LMM, we considered a second eQTL dataset of the same cell type [14] to replicate individual associations out-of-sample. In contrast to within-sample validations, external confirmation of eQTLs rules out the possibility of biased results and technical artefacts. To define a conservative set of likely true eQTL genes in the validation study, we used a standard LMM to test for associations between the locus 12q15 and 9106 genes for which expression data were available in both studies (*p* < 0.01, Bonferroni adjusted). We then assessed the *trans* eQTLs discovered on the Cardiogenics study by evaluating the proportion of eQTLs that could be replicated in the validation set (Figure S17 in Additional file 1). Encouragingly, the validation rate of GNet-LMM was notably higher than for any other method and in particular GNet-LMM was the only approach with a better replication rate than a standard LMM.

## Conclusion

We have here described GNet-LMM, an efficient statistical approach to increase power in *trans* genetic analyses of gene expression levels. The model reconstructs local gene regulatory networks on a genome-wide scale to identify and account for exogenous genes that either tag confounding factors or explain biological co-regulation. To do so, the model builds on well-established principles from causal reasoning [37]. Our approach is also related to Mendelian randomization [48, 49] and methods to reconstruct (small) directed acyclic graphs for multi-trait GWAS [50]. Importantly, and unlike existing methods for eQTL discovery, GNet-LMM is a local method that circumvents reconstructing whole-genome networks [36, 51], allowing applications to larger datasets. The approach is also distinct from feature selection methods used to select genetic covariates [35], as the networks reconstructed by GNet-LMM are directed, which is central to define exogenous factors. Using the principles of V-structures testing, our approach locally scans all gene triplets in which at least one gene has a strong genetic anchor. This approach identifies a typically small number of exogenous genes (Figure S22 in Additional file 1), which can be efficiently accounted for within the LMM framework by using low-rank updates of a random effect covariance (see “Materials and methods”). Other more global discovery procedures, such as the PC algorithm [52, 53], could be considered as an alternative. However, they come at the cost of an increased computational burden, in particular for dense networks.

Using simulations, we have shown that inference of local directed networks can be used to increase power of eQTL mapping in a wide range of settings, including different genetic architectures and types of confounding (Fig. 2; Figure S6 in Additional file 1). Our results also provide new insights into the limitations of existing methods based on PCA or factor analysis. In particular in the regime of pleiotropic genetic signals, e.g. due to regulatory hotspots, such approaches can skew eQTL detection power towards single-SNP–single-gene associations. Notably, GNet-LMM is neutral regarding the presence of regulatory hotspots. The method neither assumes that *trans* eQTLs form hotspots (e.g. [27]) nor suffers from explaining away genetic signals that result from such master regulators if they exist (Fig. 3). Although we do not consider this here, GNet-LMM[cis] implicitly establishes a causal chain between the variant, an intermediate *cis* anchor and the focal *trans* gene. The analysis of mediating causal genes is of considerable interest in itself and methods to identify mediating genes, such as the Trigger algorithm [20] and related methods [54, 55], have been described elsewhere. It is also worth noting that accounting for exogenous factors can be beneficial even in the absence of confounding, where exogenous genes capture regulatory dependencies of gene networks (Fig. 3a).

Although we demonstrated that GNet-LMM is robust in different analyses, the method is not free of limitations. One key requirement for the model to work is the presence of mediating genes that can be leveraged as an anchor to identify V-structures (Fig. 1a). While *trans* associations with a *cis* anchor are arguably among the most plausible and relevant mechanisms for *trans* effects [21], there are other ways in which genetic variants can affect expression levels of downstream genes, such as epigenetic modifications or transcription initiation [56]. In principle, any gene with an established genetic effect can be used to test for V-structures, as illustrated by GNet-LMM[trans], where strong *trans* associations are used as an anchor to identify V-structures and exogenous genes (Figure S14 in Additional file 1). However, the mechanisms of *tran*s effects that are *cis* mediated are much better understood, and the additional search for *trans* associations increases the computational cost and entails additional V-structure tests. Thus, GNet-LMM[cis] may be more relevant in most practical settings. A second limitation is the need to set additional model parameters and significance thresholds. We have found that overall GNet-LMM is remarkably robust to these parameters (“Materials and methods”; Figures S18, S19, and S20 in Additional file 1); however, data from new platforms and much larger forthcoming eQTL studies may benefit from further refinements.

Finally, it is important to note that GNet-LMM is complementary to existing methods that have been designed for the analysis of *cis* eQTLs. Because anchor genes do not exist in this instance, GNet-LMM cannot discover exogenous genes and hence existing methods such as SVA [57], PEER [11], PANAMA [12] or ICE [10] remain the method of choice for *cis* eQTL mapping.

In summary, we have proposed a simple covariate selection approach for *trans* eQTL mapping that exploits local directed gene regulatory networks to identify exogenous genes. We provide new insights into limitations of existing methods, in particular when including covariates based on PCs in genetic models. GNet-LMM increases power compared with previous methods. In addition, we observe that even in the absence of confounding factors, it is beneficial to account for exogenous genes that capture regulatory context. The statistical building blocks we have used in this study could be adapted to other more complex analyses, including joint modelling of gene expression profiles from multi-tissue eQTL studies [9, 15, 58]. In such cross-tissue analyses GNet-LMM could help to improve our understanding of the tissue specificity of *trans* genetic effects.

## Materials and methods

### GNet-LMM algorithm

Let *N* be the number of samples, *F* the number of markers and *T* the number of genes with observed gene expression levels. Let **Y** denote the gene expression matrix and the genotype matrix is denoted by **X**. Furthermore, the expression levels of the *t*-th gene across individuals are denoted **y**_t_ and the genotypes of the *f*-th marker are indexed as **x**_f_.

GNet-LMM builds on the principal that conditioning on exogenous factors (here genes) can increase power to detect *trans* associations. To model the expression level of a *focal* gene C, the method tests for associations with genetic variant of interest (SNP A) while accounting for exogenous factors (gene B), which are defined by (i) having a causal effect on the *focal* gene C *and* (ii) being unrelated to the causal path that links the genetic effect of the variant (SNP A) to the *focal* gene (Fig. 1a).

Specifically, for each SNP A–gene C pair to be tested, we first search for triplets of genes that form a so-called V-structure (gene A - > gene C < - gene B), where both genes A and B have a causal effect on the focal gene C (Fig. 1a). A set of basic statistical dependencies can be used to identify these structures from the expression data itself, where A–C and B–C are tested for dependence and A and B are required to be independent, but become dependent after conditioning on gene C (see e.g. [37] for more details):

1 $$ dep\left({\boldsymbol{y}}_A,{\boldsymbol{y}}_C\right) $$

2 $$ dep\left({\boldsymbol{y}}_{B,},\ {\boldsymbol{y}}_C\right) $$

3 $$ ind\left({\boldsymbol{y}}_A,{\boldsymbol{y}}_B\right) $$

4 $$ dep\left({\boldsymbol{y}}_A,{\boldsymbol{y}}_B\ \Big|\ {\boldsymbol{x}}_C\right) $$

5 $$ dep\left({\boldsymbol{x}}_A,\ {\boldsymbol{y}}_A\right) $$

6 $$ ind\left({\boldsymbol{x}}_A,{\boldsymbol{y}}_B\right) $$

Here, *dep* denotes a statistical dependency criterion and *ind* corresponds to statistical independence and the symbol | denotes a conditional test. The vector **y**_A_ denotes the expression level of gene A and similarly **y**_B_ and **y**_C_ denote expression vectors for genes B and C, respectively. **x**_A_ denotes the genotype vector of the variant to be tested, for which we consider a conventional (0,1,2) encoding.

The additional independence test between SNP A and gene B (Eq. 6) is not required to identify gene A - > gene C < - gene B V structures. This condition is included to rule out the possibility that gene B is itself associated with the variant of interest, which can cause synthetic associations [33].

To define a computationally efficient strategy and to increase robustness, GNet-LMM restricts gene A to anchor genes with either a *cis* or *trans* association to the SNP (hence, the additional dependence requirement; Eq. 5). This condition substantially reduces the search space, thereby reducing the otherwise quadratic effort to consider all pairs of genes A and B that could form valid V-structures (see section on runtime below). Similar search strategies have previously been considered to identify mediating genes; see for example [20]. We here denote these models GNet-LMM[cis] and GNet-LMM[trans], respectively, where GNet-LMM[cis] requires a *cis* regulatory link between SNP A and gene A and GNet-LMM[trans] considers *cis* or *trans* associated genes A as anchors to test for V-structures. The algorithm proceeds by conditioning on all genes B that satisfy the V-structure criterion for a given (SNP A, gene C) pair.

**Implementation details:** The GNet-LMM algorithm can be broken down into three steps.

1. Perform an initial *genome-wide* eQTL scan, testing for associations between all variants and the expression levels of all genes, resulting In a TxF-dimensional matrix of *p* values.
  Runtime: O(N^3^ + FTN^2^).
2. For each gene C, search for gene pairs (**y**_A_, **y**_B_) that form a V-structure with the focal gene **y**_C_ (gene A - > gene C < - gene B).
  Runtime: O(T^2^N + k N), where computing the marginal correlation takes O(T^2^N) time and *k* is the number of partial correlation coefficients to be computed. To reduce computations, we only compute the partial correlation coefficient if the marginal correlations satisfy the first three V-structure criteria (Eqs. 1, 2, and 3) and gene A has a SNP anchor (Eq. 5).
3. Update the association *p* value for all (**x**_A,_**y**_C_) marker-gene combinations by conditioning on the set of genes **y**_B_ that satisfy the V-structure criterion, where **x**_A_ is a *cis*/*trans*-anchor to gene **y**_A_ and (**y**_A_, **y**_B_) build a V-structure with gene **y**_C_.
  Runtime: O(sN^2^R + mNR^2^), where *s* is the number of unique conditioning sets, *m* is the total number of associations to be updated and R is the number of genes in the conditioning set. Although R varies from test to test, it is bounded in practice (Figure S22 in Additional file 1) and our software implementation allows specifying an upper limit for the permitted rank R.

In the following, we give a more detailed description of each step:

**Step 1 (association scan):** We employ a standard LMM [59–61] to test for associations between variant **x**_A_ and gene **y**_C_:

7 $$ {\boldsymbol{y}}_C \sim N\left({\boldsymbol{x}}_A\beta, {\sigma}_{bg}^2{\boldsymbol{K}}_{bg}+{\upsigma}_{\mathrm{n}}^2\mathbf{I}\right) $$

Here, ***K***_*bg*_ denotes the random effects covariance matrix, σ_bg_^2^ denotes the variance of the random effect term, σ_n_^2^ denotes the magnitude of the noise and **I** is the identity matrix, which corresponds to the assumption of iid measurement noise. Additional fixed effect covariates are omitted for brevity but can be included analogous to the effect of the variant ***x***_*A*._ The background covariance matrix ***K***_*bg*_ can be flexibly chosen, such as a global kinship matrix to model relatedness [62] or a local kinship matrix to model *cis* effects that act on the focal gene [19]. In the experiments, we use the realized relationship matrix [62] as background covariance, thereby adjusting for population structure. As initially proposed in the EMMA-X approximation [63], the ratio between the noise and background covariance is fit once on the null model using maximum likelihood, and kept fixed for each test. We use the fastLMM algorithm [60] as implemented in LIMIX [64] for all model fits and association tests.

**Step 2 (detecting V-structures):** A V-structure can be uniquely identified by the set of independence tests described in Eqs. 1, 2, 3, and 4. In addition, we require that gene **y**_A_ has a strong *cis*-association to **x**_A_ (Eq. 5), whereas **y**_B_ is tested for independence to the marker **x**_**A**_ (Eq. 6). The latter criterion ensures that the potential exogenous factor **y**_B_ is not in association with the variant of interest, which otherwise may lead to spurious associations (Figure S3a in Additional file 1).

Following the approach taken in [65], we employ a standard correlation test to assess dependence between genes and test if the *p* value is larger than a pre-defined threshold to assess independence of gene **y**_A_ and gene **y**_B_. Although this independence criterion is not a well-defined statistical test (the null-distribution is not defined over the null but from the alternative distribution), this approach has been shown to work well in practice, e.g. [55]. When testing for dependence between a gene and a SNP, we use the association test (Eq. 7) to test for independence, again assuming that independence is present if the *p* value is larger than a certain threshold. Explicit independence tests such as the Hilbert-Schmidt independence criterion [66] could also be considered, although these are computationally more demanding and hence were not used in this study.

**Step 3 (update association scan):** Following the exhaustive search of V-structures (gene A - > gene C < - gene B), we use an extended LMM approach as in Eq. 7 to condition on the expression of all exogenous genes **y**_B_ that satisfy the V-structure criterion for **x**_A_ and gene **y**_C_:

$$ {\boldsymbol{y}}_C \sim N\left({\boldsymbol{x}}_A\beta, {\sigma}_{bg}^2{\boldsymbol{K}}_{\boldsymbol{bg}}+{\sigma}_{exo}^2\ {\displaystyle {\sum}_{B\ \in\ {L}_{exo}}{\boldsymbol{y}}_B{\boldsymbol{y}}_B^T+{\sigma}_n^2\boldsymbol{I}}\right). $$

The parameter additional parameter *σ*_*exo*_^2^ determines the variance explained by the set of exogenous genes and *L*_*exo*_ denotes the set of identified exogenous genes. Since conditioning sets typically contain only a small number of genes (Figure S22 in Additional file 1), we use a low-rank LMM implemented in the mtSet method [67] to test for associations similar to the approach described in Step 1. This approach allows fitting this model efficiently to large datasets. The implementation of GNet-LMM allows limiting the maximum number of genes in the conditioning set. This is approximation to the full model can be used to further reduce the overall runtime, if desired (see “Implementation details”).

**Parameters and (in)dependence thresholds:** We used the following strategy for setting the thresholds for dependence and independence tests:

- Two genes are (conditionally) dependent if they have an adjusted *p* < 0.01.
- Two genes are independent if they have a *p* ≥ 0.1.
- A SNP is associated with its *cis*-gene if the adjusted *p* < 0.05*.*
- A SNP is independent of the exogenous gene if the *p* ≥ 0.1.

We have used these settings in all experiments except for the basic simulation (Figure S1, S2, and S3 in Additional file 1), where due to the small number of genes more stringent criteria were employed. In this setting, we considered a threshold of *p* < 0.001 for calling genes correlated, *p* > 0.1 for calling genes independent and *p* < 0.001 for calling *cis* associations. We also investigated the impact of changing the specific parameter values, finding that GNet-LMM is overall robust with respect to the specific threshold used (Figure S18, S19, and S20).

**Statistical calibration:** It is important to ensure that the GNet-LMM yields calibrated test statistics on the null distribution, in particular for tests where V-structures have been identified and hence additional genes for conditioning are used. We considered two experiments to verify calibration empirically. First, we confirmed that *p* values are calibrated if a V-structure is evoked by confounding effects, e.g. if a hidden common cause induces correlations between gene A and gene C without the existence of a causal relationship between SNP A and gene C (Figure S3b in Additional file 1). Secondly, we verified that no V-structure is detected if the gene to be conditioned on (gene B) is also associated with the SNP of interest (Eq. 6), which avoids the possibility of synthetic associations (Figure S3a in Additional file 1).

**Runtime complexity:** On the Cardiogenics dataset, computing all gene–gene correlations required 370 s. Running the initial association scan with LMM took approximately 18 s per gene and updating the results took, on average, 86 s when considering *cis*-anchors and 105 s when using *trans*-anchors. Empirical runtimes were evaluated using one single core of an Intel Xeon CPU E5-2670 2.60 GHz processor. The GNet-LMM software implementation allows parallelizing the core operations on a large compute cluster.

### Implementation of alternative methods

In the experiments, we compared GNet-LMM to representatives of alternative methods, either based on random effect models or approaches that use PCs to account for confounding. As a baseline, we considered a standard LMM with a realized relationship matrix [10] as random effect covariance matrix (LMM). ICE-LMM denotes a LMM where a second random-effect covariance was estimated from the empirical gene expression covariance matrix [10, 26]. In PC-LMM, the first n principal components were accounted for as a random effect term. Similarly, in PCselect-LMM, the same approach was used but excluding PCs in association with at least one variant (qval < 0.2). For both PC-LMM and PCselect-LMM, the number of PCs was determined in the range (10,20,30,40,50), maximizing the total number of *trans*-associations, as previously considered in [18, 22]. All methods were implemented within the LIMIX framework [64].

### Simulation study

Simulated datasets were generated by using synthetic gene expression levels and genotypes from chromosome 20 of 1000 Genomes individuals of European populations (379 individuals) [38]. To avoid possible biases in the evaluation of methods on simulated data when causal and non-causal variants are in strong linkage disequilibrium, we reduced the variant set to limit local linkage disequilibrium to a maximum of r^2^ = 0.9 (within windows of size 50, step length 5). As an additional filter, we discarded rare variants with a minor allele frequency of less than 5 %, resulting in 4030 quasi-independent common variants. All simulation experiments were performed on the reduced dataset.

**Basic simulation:** We studied the following four small gene–gene networks:

- Power (Figure S1 in Additional file 1):
  - SNP A → Gene A;
  - Gene A, Gene B → Gene C,
  - Gene C → Gene D

In the default setting, the *cis*-anchor of gene A explains between 10 % and 20 % of the variance, and each regulating gene explains between 10 % and 20 % of the variance.

- Direct effect (Figure S2a in Additional file 1):
  - SNP A → gene A, gene C;
  - Gene A, Gene B → Gene C

We use the same default parameters as before, but introduce an additional parameter α regulating how the variance explained by SNP A is divided between gene A and gene C.

- *Trans*-effects (Figure S2b in Additional file 1):
  - SNP A → Gene A, Gene D;
  - Gene A, Gene B, Gene D → Gene C

We use the same default parameters as before, but introduce an additional parameter α regulating how the variance explained by SNP A is divided between Gene A and Gene D.

- Confounding (Additional file 1: Figure S3):
  - SNP A → Gene A, (Gene B);
  - Confounder A → Gene A, Gene C;
  - Confounder B → Gene B, Gene C

The *cis*-anchor of gene A explains between 10 % and 20 % of the variance, and the confounding effects explain together 50 % to 70 % of the gene’s variability.

The gene expression levels are simulated as a linear function of incoming edges, the *cis* SNP and noise. In each experiment, we assessed the statistical power of alternative methods to detect simulated *trans* association between SNP A and gene C. Each experiment was repeated 1000 times.

**Power comparison:** Next, we studied gene–gene networks consisting of 100 genes, considering alternative network topologies, as well as different types and the strengths of confounding. Gene expression levels were simulated as a linear additive combination of effects due to incoming edges, the *cis*-SNP, noise and confounding factors. We considered two network architectures; first, sparse and unstructured networks in which edges are randomly drawn from a Bernoulli distribution and second star-shaped and structured networks with nine randomly selected hub genes that regulate between 20 % and 50 % of all other genes. In addition, we simulated a varying number of confounding factors, and randomly assigned genes to be affected by confounding factors. The regulatory weights were drawn from a mixture of two normal distributions, with a mixture coefficient of 0.5. The mean of both components was set to ±1 and the standard error for each component is 0.1. This prior design helps to ensure that simulated edges have non-zero weights, with equal proportions having positive and negative effects.

To consider alternative genetic designs, we altered the variance explained by the *cis*-SNP (0.0,0.05,**0.10**,0.15,0.20), the variance explained by the networks (joint effect of confounding and genetic network; 0.0,0.5,0.60,7,**0.8**,0.9), the proportion of variance explained by confounding factors (0.0,**0.2**,0.4,0.6,0.8,1.0), the number of confounding factors (0,1,2,**3**,4,5), and the expected number of confounders per gene (0,0.5,**1**,3,5). The default settings are marked in bold. For further details on the simulation approach, see Supplementary methods in Additional file 1.

**Evaluation:** SNP–gene associations were ranked in ascending order by their *p* value. An association between SNP A and gene C was considered a true positive if gene A is a direct regulator of gene C and the associated variant was close to the simulated *cis* anchor (SNP A, ±2 kb). In order not to confound the *trans* eQTL analysis, we ignored putative *cis* associations within a window of size ±5 Mb. We defined power as the area under the receiver operating characteristic (ROC) curve for a false positive rate below 5 %.

### Mouse dataset

We considered gene expression levels measured in hippocampus tissue of 468 heterogeneous stock mice [40]. The dataset contained 12,545 genetic markers and expression levels for 19,892 genes. In order to facilitate the evaluation of *trans* eQTLs, we considered the set of 3740 genes that could be linked to at least one Reactome pathway. We considered associations as Reactome-consistent if the associated variant and the focal gene were linked by at least one pathway. For this mapping of variants to genes, we considered all genes with a transcription start site located within ±2 Mb around the variant. We excluded all associations that were likely due to *cis* effects (±20 Mb) from the analysis. We selected 20 factors for PC-LMM and PCselect-LMM maximizing the number of *trans* associations (see Figure S21 in Additional file 1 for alternatives).

### Cardiogenics transcriptomic study

We analysed the data from the Cardiogenics Transcriptomic Study [44, 45]. We restricted the analysis from the 758 individuals to the 395 samples from the Cambridge cohort, to reduce batch effects and differences in disease status (the Cambridge cohort was exclusively from healthy individuals). Out of these, 376 individuals passed quality control steps as in the primary analysis of the data [44, 45]. Requiring a minor allele frequency of at least 0.05, this resulted in 502,378 variants for analysis. We considered matching expression levels for 15,340 probes. Individual expression levels were quantile normalized to unit variance normal distribution. Subsequently, we regressed out age and gender and again used quantile normalized of the residuals to a standard normal distribution. We excluded associations that are likely *cis* acting (±2 Mb) from the analysis. We selected ten hidden factors for PC-LMM, and PCselect-LMM, again maximizing the number of *trans* associations (Figure S14 in Additional file 1).

### Genetics of gene expression in primary human immune cells study

We validated the results from the Cardiogenics Transcriptomic Study [68] in 414 independent monocyte expression arrays in an independent monocyte eQTL dataset [18]. eQTL replication was assessed on a set of 9106 overlapping probes, considering eQTLs downstream of the locus 12q15.

### Software

A standalone python implementation of GNet-LMM, including examples and use cases, is available under an Apache licence at https://github.com/PMBio/GNetLMM. The software builds on software components available in mtSet [67] and LIMIX [64].

### Data access

The mouse dataset is publicly available on Array Express under the accession number E-MTAB-88. Published data from the cardiogenics and the Genetics of gene expression in primary human immune cells study have been deposited at the European Genome-phenome Archive (EGAS00001000411 and EGAS00000000109).

### Ethics approval

Ethics approval was not needed for this study.

## Additional file

Additional file 1:
**Supplementary methods and supplementary figures.** (PDF 5596 kb)

## Abbreviations

eQTL: Expression quantitative trait locus
GNet-LMM: Gene network linear mixed model
GWAS: Genome-wide association studies
LMM: Linear mixed model
PC: Principal component
PCA: Principal component analysis
ROC: Receiver operating characteristic
SNP: Single nucleotide polymorphism
