# Supplementary material for: Modelling local gene networks increases power to detect trans-acting genetic effects on gene expression
Source: Genome Biol. 2016 Feb 24;17:33. doi: 10.1186/s13059-016-0895-2 (PMC4765046; doi:10.1186/s13059-016-0895-2)
Supplement: Additional file 1: — Supplementary methods and supplementary figures. (PDF 5596 kb) [file 13059_2016_895_MOESM1_ESM.pdf]

Supplementary Material: Modeling local gene networks increases power to detect trans-acting genetic effects on gene expression

Barbara Rakitsch, Oliver Stegle

Contents

1 Supplementary methods **3**
1.1 Basic simulation study 3
1.2 Power Simulations 4

2 Supplementary results **4**

List of supplementary figures

S1 Power comparison across different settings for the basic simulation. 5
S2 Power comparison for direct, *cis*- and *trans*-mediated SNP effects. 6
S3 GNet-LMM is robust to confounding factors. 7
S4 Graphical Model of the data generation process for simulations that include confounding effects. 8
S5 Principal components capture confounding factors and genetic master regulators. 8
S6 Power comparison of alternative methods across different simulation experiments. 9
S7 Genomic control on the mouse dataset. 10
S8 The latent factors learnt by PC-based methods are associated with putative *trans* hotspots. . . . 11
S9 Reactome enrichment analysis of eQTLs identified on the mouse dataset. 12
S10 Quantile-quantile plots on the mouse dataset. 13
S11 Number of *cis* associations retrieved by alternative methods for varying p-value thresholds on the mouse dataset. 14
S12 Calibration of alternative methods on the Cardiogenics dataset. 14
S13 Number of retrieved *cis* associations on the Cardiogenics dataset. 14
S14 Number of *trans* associations associations retrieved by alternative methods on the Cardiogenics dataset. 15
S15 Number of *trans*-genes identified downstream of the marker rs6581889 in the LYZ region for varying cutoff values on the Cardiogenics dataset. 15
S16 String Network for genes that are associated with the LYZ region ($pv < 0.01$, Bonferroni adjusted) on the cardiogenics dataset. 16
S17 Validation of genes that are associated with the LYZ region on the Cardiogenics dataset using an independent Monocyte eQTL study. 17
S18 Consistency of GNet-LMM p-values for varying *cis*-thresholds on the mouse dataset. 17
S19 Consistency of GNet-LMM p-values for varying adjusted *ind*-thresholds on the mouse dataset. . 18
S20 Consistency of GNet-LMM p-values for varying adjusted *assoc*-thresholds on the mouse dataset. 18
S21 Selection of the number of principle components for PC-based methods on the mouse dataset. . . 19
S22 Distribution of the number of genes in the conditioning set for the Cardiogenic dataset. 19

1 Supplementary methods

1.1 Basic simulation study

Power

We first studied a small gene-gene network to show the benefits of our model. The network consists of four genes A, B, C and D, with the following topology:

$$A, B \rightarrow C \tag{1}$$
$$C \rightarrow D \tag{2}$$

Gene A has a cis-anchor explaining between 10% and 20% of the variance and each regulating gene explains between 10% and 20% of the variance. The remaining variance goes into i.i.d. noise. We are interested in finding *trans* associations and for simplicity here concentrate on the association between SNP A and gene C.

Next, we keep all but one parameter fixed, and vary

- the variance explained by SNP A
- the variance of the regulating effect A $\rightarrow$ C
- the variance of the regulating effect B $\rightarrow$ C

in the interval $\{0.00, 0.01, 0.05, 0.10, 0.15, 0.20, 0.25\}$. For each setting, 1,000 independent datasets were simulated.

Confounding

Next, we studied a small gene-gene network in which conditioning on the wrong gene leads to false positives. The networks consists of three genes A, B and C, and two confounding factors H_A, H_B:

$$H_A \rightarrow A, C \tag{3}$$
$$H_B \rightarrow B, C \tag{4}$$

Gene A and Gene B have the same *cis* anchor explaining in total between 10% and 20% of the variance andand the confounding effects explain together 50% to 70% of the gene$\sqrt{}$is variability.. The remaining variability goes into i.i.d noise. We are interested in testing if GNet-LMM calls V-structures that lead to false positives between SNP A and Gene C.

We keep all parameter fixed and

- use the same *cis* SNPs for gene A and gene B to test for synthetic associations
- use different *cis* SNPs for gene A and gene B to examine the effects of confounding in general

Each setting is repeated 10000 times.

Parallel

In the following, we study a small gene-gene network in which a SNP has a direct and an indirect (cis-mediated) effect on the target gene. The network consists of three genes A,B and C having the following structure:

$$A, B \rightarrow C \tag{5}$$

Gene A has a *cis* anchor explaining between 10% and 20% of the variance and each regulating gene explains between 10% and 20% of the variance. The remaining variance goes into i.i.d. noise. We are interested if GNet-LMM improves power compared to a standard LMM depending depending if the association is *cis* mediated or not. For this, we introduce an additional parameter α, where $(1-\alpha)^2$% of the variance explained by SNP A goes to Gene A and α^2 goes directly to gene C. We let α vary in $\{0.2, 0.4, 0.6, 0.8, 1.0\}$ and repeat each setting 1000 times.

Trans

Finally, we study a small gene-gene network in which a SNP effect is mediated by a *cis* and by a *trans* effect on gene. The network consists of four genes A,B, C and D having the following structure:

$$A, D, B \rightarrow C \quad (6)$$

Gene A has a *cis* anchor and Gene D has a *trans* anchor with the same SNP. In total, the SNP explains between 10% and 20% of the variance and each regulating gene explains between 10% and 20% of the variance of gene C. The remaining variance goes into i.i.d. noise. We are interested if GNet-LMM improves power compared to a standard LMM depending if the association is *cis* mediated or not. For this, we introduce a new parameter α, where $(1-\alpha)^2$% of the variance explained by SNP A is mediated by Gene A and α^2 is mediated by Gene D. We let α vary in $\{0.2, 0.4, 0.6, 0.8, 1.0\}$ and repeat each setting 1000 times.

1.2 Power Simulations

Each gene is a linear function of all in-coming edges, the *cis*-SNP and noise:

$$Y_{i,gene} = \sum_{j,A_g[i,j]=1} Y_j w_{i,j} \quad (7)$$
$$Y_{i,cis} = X_{i,cis} w_{i,cis} \quad (8)$$
$$Y_i = \sigma_{SNP} Y_{i,cis} + \sigma_{gene} Y_{i,gene} + \epsilon_i, \quad (9)$$

The matrix A_g is the adjacency matrix where $A_g[i,j] = 1$ iff gene j causes gene i. The simulated networks contained 100 genes.

The gene weights are drawn from a mixture of two normal distribution, where the mixture coefficient is 0.5 and the means are ± 1 and the standard error from each component is 0.1. By using this prior, we ensure that all in-coming edges have roughly the same impact.

Sparse Network We draw a directed edge between gene i and gene j with probability 5%. In addition, we require that $i < j$ to ensure that there are no loops in the network and allow for no more than 5 in-coming edges.

Star Network Select the first 9 genes as hubs. Each hub regulates between 20% and 50% of the genes. No hub is regulated by another hub.

Confounding Factors

$$Y_{i,cis} = X_{i,cis} w_{i,cis} \quad (10)$$
$$Y_{i,gene} = \sum_{j,A_g[i,j]=1} Y_{i \setminus conf} w_{i,j} \quad (11)$$
$$Y_{i,conf} = \sum_{j,A_c[i,j]=1} U_j w_{i,j} \quad (12)$$
$$Y_{i,network} = (1-\alpha) Y_{i,gene} + \alpha Y_{i,conf} \quad (13)$$
$$Y_i = \sigma_{SNP} Y_{i,cis} + \sigma_{network} Y_{i,network} + \epsilon_i \quad (14)$$

We first simulate genes according the gene network. Afterwords, we add confounding to the genes.This should resemble technical confounding that is not propagated by the gene-gene network. We again use a mixture of two normal distribution for the weights of the confounding factors.

We vary the following parameters:

- the variance of the SNP in $\sigma^2_{SNP} \in \{0.0, 0.05, \mathbf{0.10}, 0.15, 0.20\}$
- the variance of the network $\sigma^2_{network} \in \{0.0, 0.3, 0.5, 0.7, \mathbf{0.8}, 0.9\}$
- the ratio between the confounding factors and the gene network $\alpha \in \{0.0, \mathbf{0.2}, 0.4, 0.6, 0.8, 1.0\}$
- the expected number of confounders per gene in $\{0, 0.5, \mathbf{1}, 2, 3\}$
- the number of confounders $\{0, 1, 2, \mathbf{3}, 4, 5\}$

The default settings are marked in bold. Each setting is repeated 30 times.

2 Supplementary results

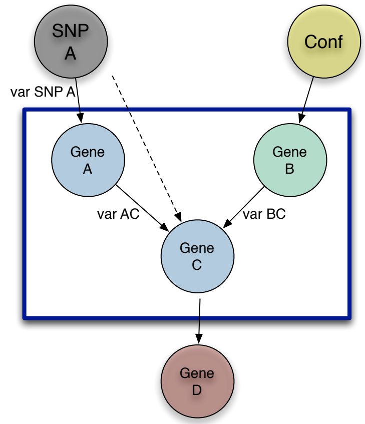

(a) network topology

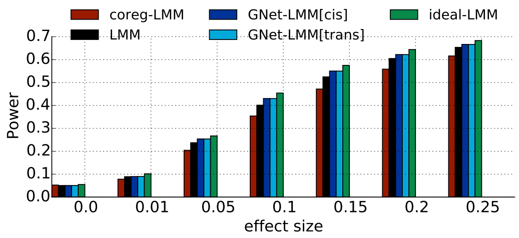

(b) varying effect of SNP A

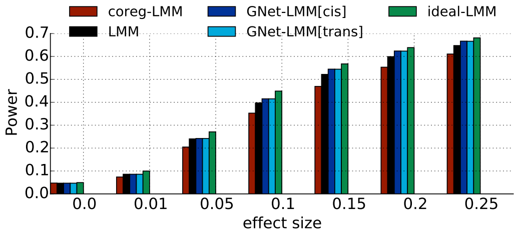

(c) varying effect of gene A

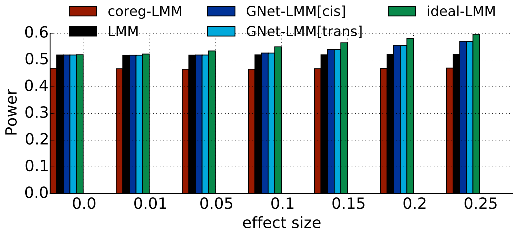

(d) varying effect of gene B

Figure S1: **Power comparison across different settings for the basic simulation.** Compared are a standard linear mixed model (LMM), GNet-LMM with *cis* and *trans* anchors as well as an LMM when conditioning on *true* exogenous genes (ideal-LMM) and an LMM when conditioning on co-regulated genes downstream of the focal gene C. (**a**) the topology of the simulated network (see main text, Methods). Considered are alternative simulation parameters, varying (**b**) the proportion of variance explained by SNP A on the mediating (*cis*) gene A, (**c**) the effect of the edge effect of Gene A on Gene C, (**d**) the variance explained by Gene B on Gene C. Across the different scenarios, the performance of GNet-LMM is close to the theoretical limit when conditioning on true exogenous genes (ideal-LMM).

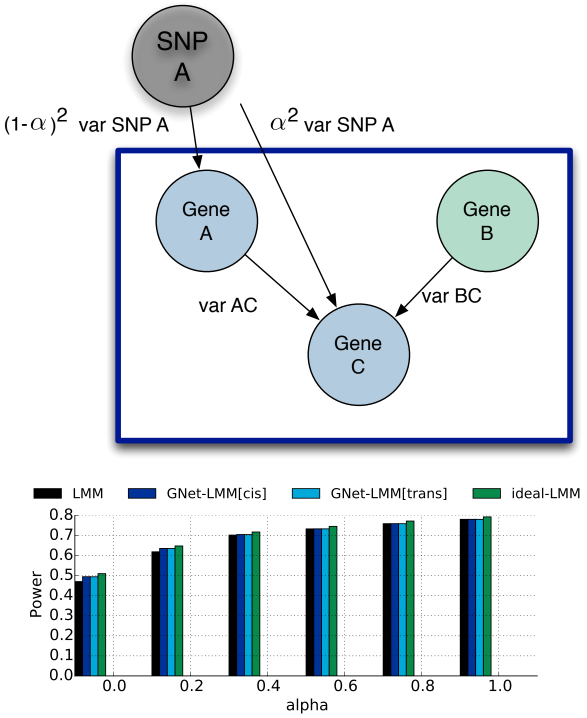

(a) direct association

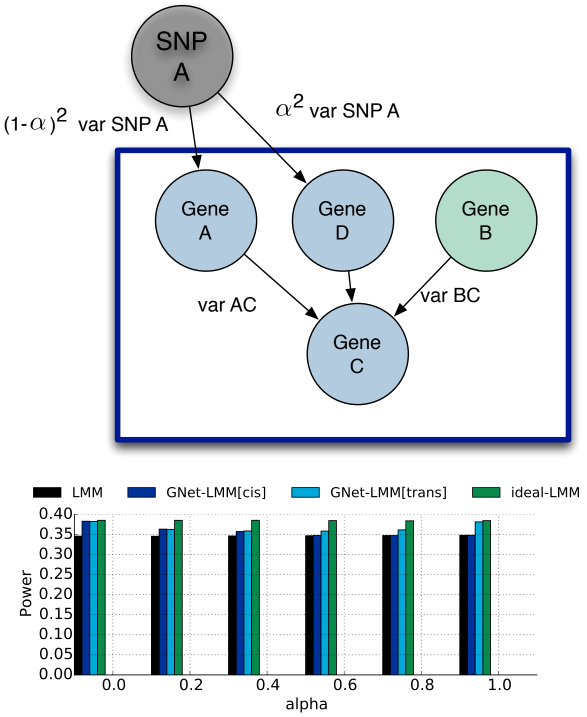

(b) trans-mediated association

Figure S2: **Power comparison for direct, *cis*- and *trans*-mediated SNP effects.** The simulation approach was analogous to results in Supplementary Figure 1, however varying the proportion of genetic effects that were intermediated by Gene A. (**a**) Altering the proportion of genetic effects that are mediated via gene A. When all signal is *cis* mediated ($\alpha = 0$) via Gene A, the power of GNet-LMM is maximized and approaches the ideal model. If the genetic effect on gene C is not *cis*-mediate, neither GNet-LMM[cis] nor GNet-LMM[trans] are able to detect V-structures and the models perform identical to a standard LMM. (**b**) Altering the proportion of variance on gene C that is mediated via *cis* (gene A) and *trans* (gene D) genes. In case that the SNP signal is mediated by a *trans* gene (Gene D), GNet-LMM[trans] is still able to identify V-structures, and hence outperforms GNet-LMM[cis].

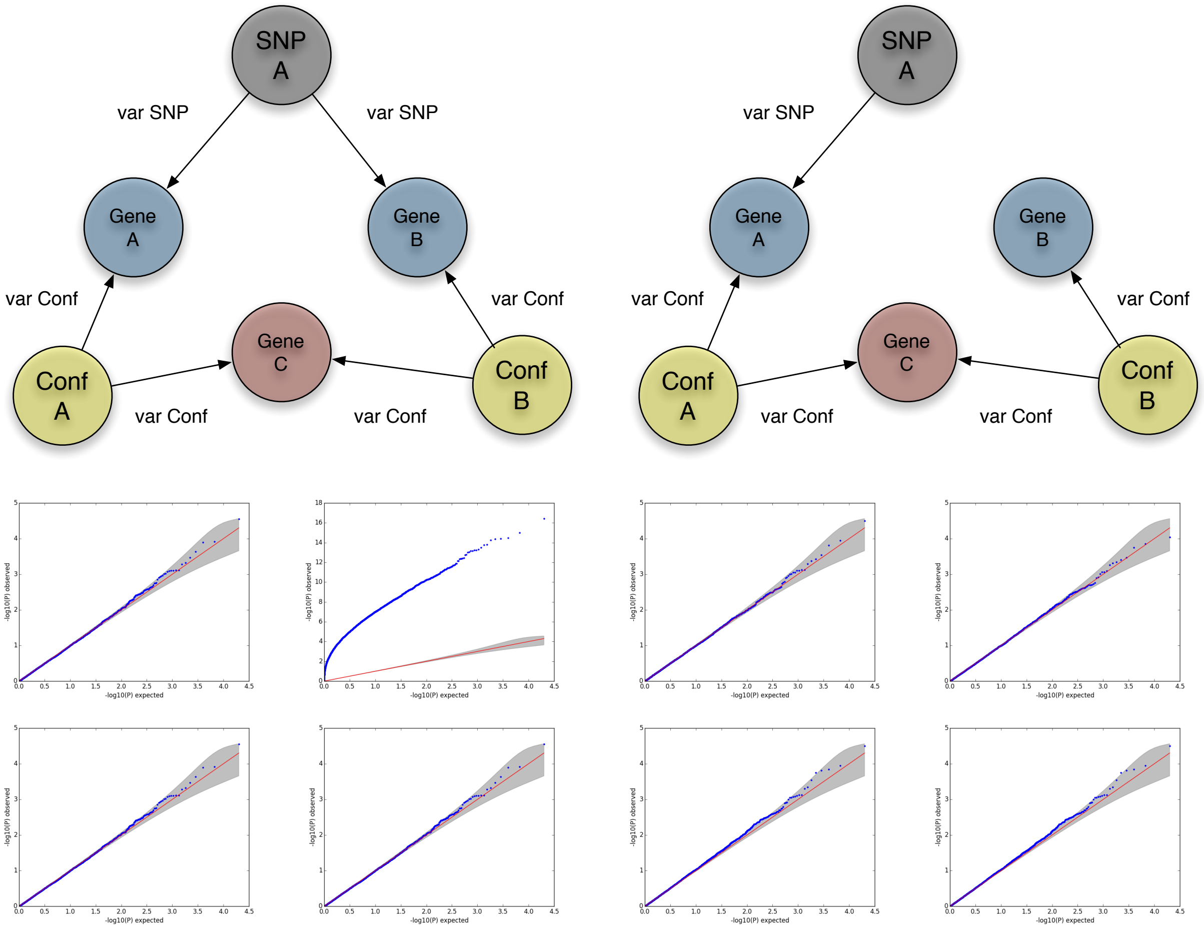

(a) synthetic association **(b)** confounding

Figure S3: **GNet-LMM is robust to confounding factors.** Shown are 1,000 simulated experiments form different specific regulatory models that address alternative scenarios of confounding. Left (**a**): If a V-structure between Gene A, Gene B and Gene C is detected, conditioning on gene B may result in synthetic associations between SNP A and gene C. This applies in particular if gene B itself is in association with the variant of interest. GNet-LMM circumvents this by additional independence tests, requiring that SNP A and gene B are independent. Shown are QQ plots from the null, where no SNP A-gene C association is present, however gene B is associated with the variant. QQ-plots from top left to right bottom: standard LMM without covariates, LMM that falsely conditions on Gene B when testing for the association, GNet-LMM[cis], GNet-LMM[trans]. Both GNet-LMM and the standard model yield calibrated tests from the null. Right (**b**): In the case that Gene B is not linked to SNP A, conditioning on gene B does not lead to synthetic associations between SNP A and gene C. QQ-plots from top left to right bottom: standard LMM with no covariates, LMM that conditions on Gene B when testing for the association, GNet-LMM[cis], GNet-LMM[trans]. The standard LMM and the LMM that conditions on Gene B are well calibrated. GNet-LMM[cis] and GNet-LMM[trans] call a V-structure in 70.17% of the experiments leading to a mixture of the former two p-value distributions that is again well calibrated.

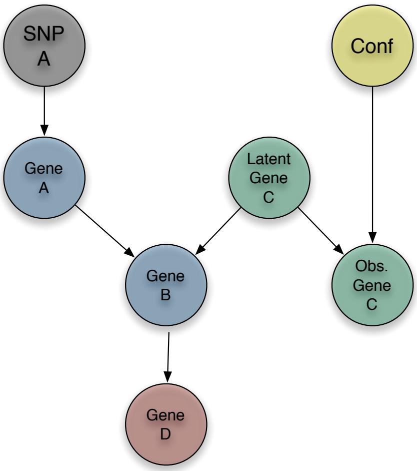

Figure S4: **Graphical Model of the data generation process for simulations that include confounding effects.** In a first step, the latent gene expression levels are simulated as function of the in-coming (latent) genes, cis-SNPs and noise. In a second step, technical noise is added to a subset of the genes leading to confounding that is not propagated along the genetic network.

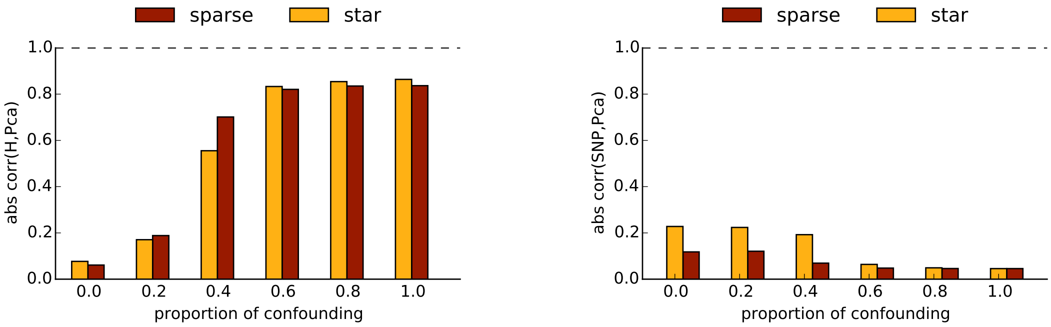

(a) corr(PC, confounder) **(b)** corr(PC, master SNP)

Figure S5: **Principal components capture confounding factors and genetic master regulators.** **(a)** Shown is the maximal absolute correlation coefficient between the first principal component on all gene gene expression levels and the known simulated confounding factors. **(b)** Shown is the absolute correlation between the first principal component and the causal SNP with the most wide-spread effect on expression (master regulator). Both statistics are shown as a function the proportion of variance explained by confounding. See methods for details on the simulation procedure.

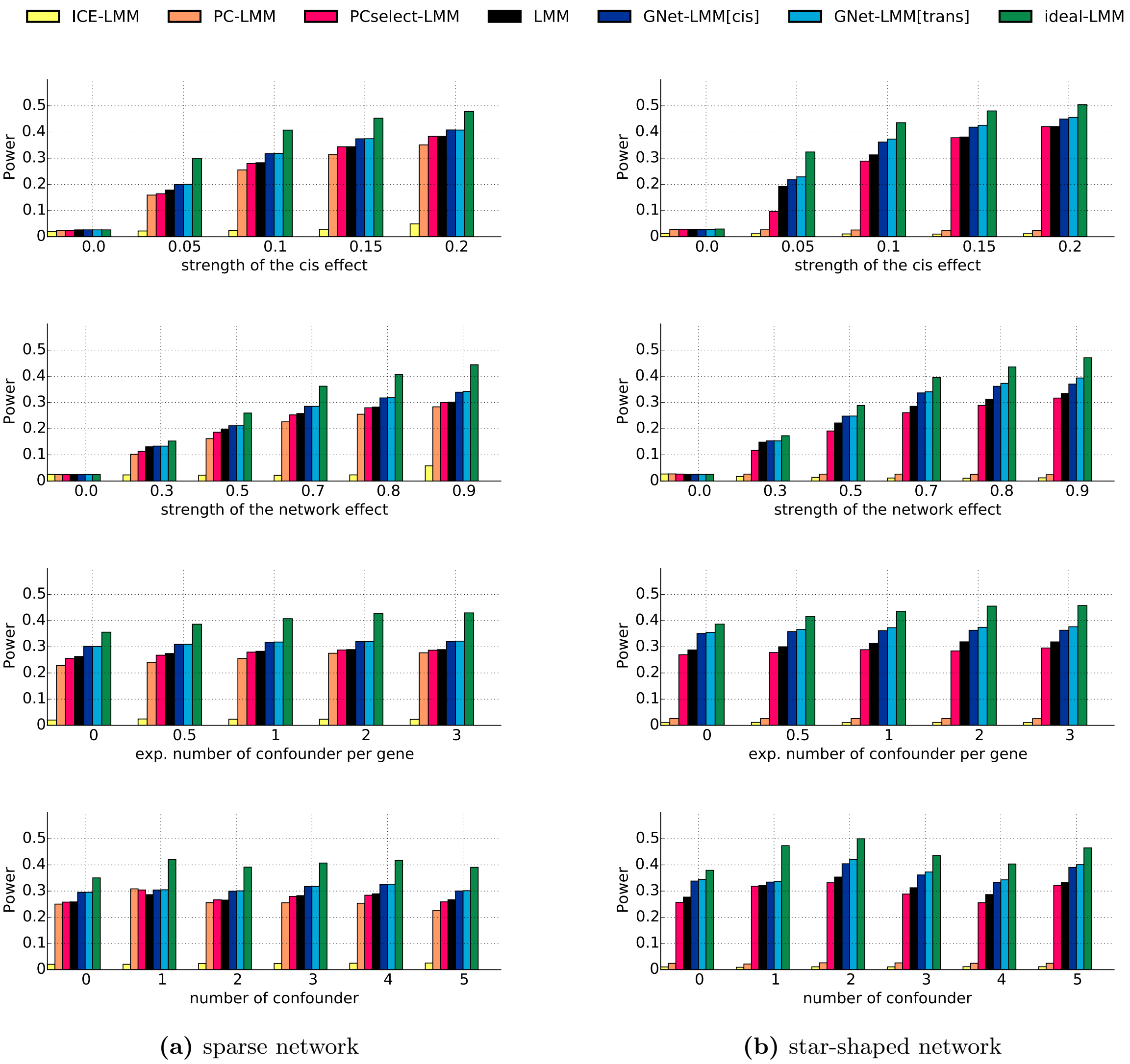

Figure S6: **Power comparison of alternative methods across different simulation experiments.** Shown is power as defined as the area under the ROC curve up to 5% false positive rate for alternative methods. Compared are ICE-LMM, PC-LMM,PCselect-LMM,GNet-LMM[cis], GNet-LMM[trans] and ideal-LMM, for alternative simulation parameters, for sparse (left) and star-shaped networks (right). Considered were different proportions of variance explained by the *cis* SNP (default: 10%), the variance explained by the network component (joint effect of the genetic and confounding network) (default: 80%), the number of expected confounders per gene (default: 1) and the total number of simulated confounders (default: 3). See methods for a detailed description of the simulation procedure.

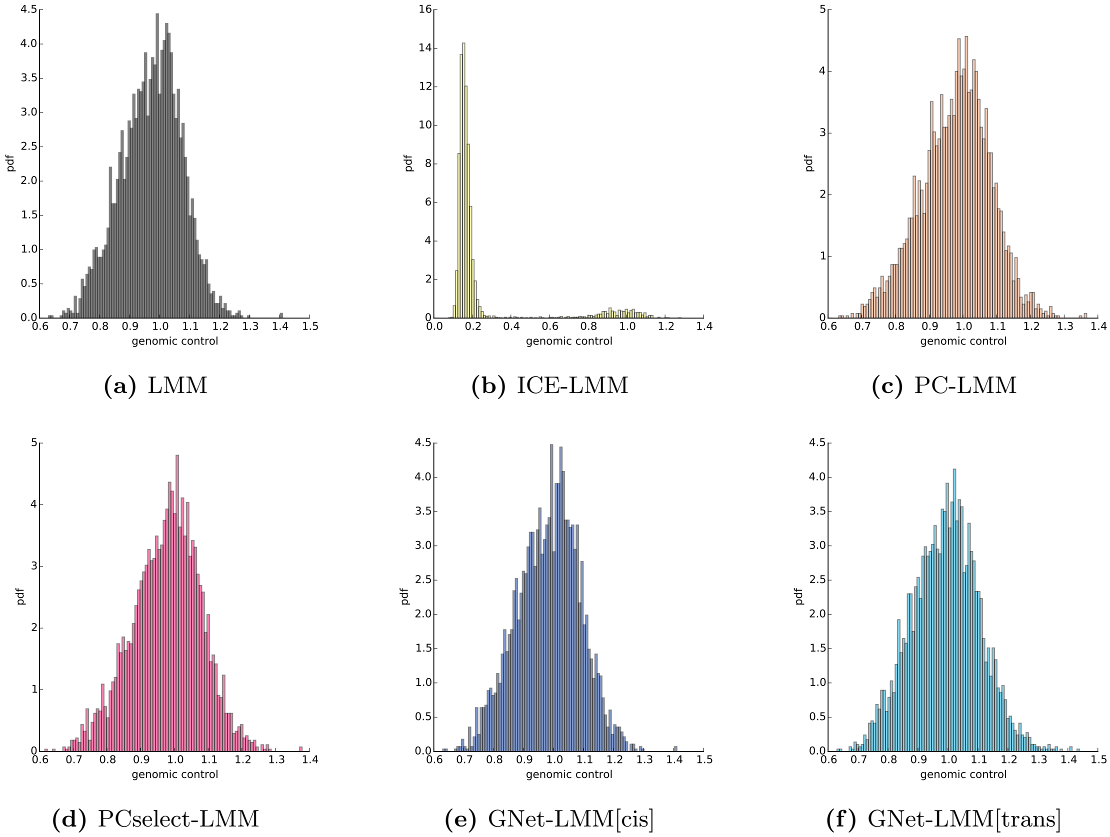

(a) LMM **(b)** ICE-LMM **(c)** PC-LMM

(d) PCselect-LMM **(e)** GNet-LMM[cis] **(f)** GNet-LMM[trans]

Figure S7: **Genomic control on the mouse dataset.** Shown are histograms of genomic control estimates across genes when considering alternative methods. Genomic control estimates were obtained from tests of *trans* associations only, where likely *cis* associations (± 20 mb around the TSS were discarded). With the exception of ICE-LMM, all methods were sufficiently calibrated.

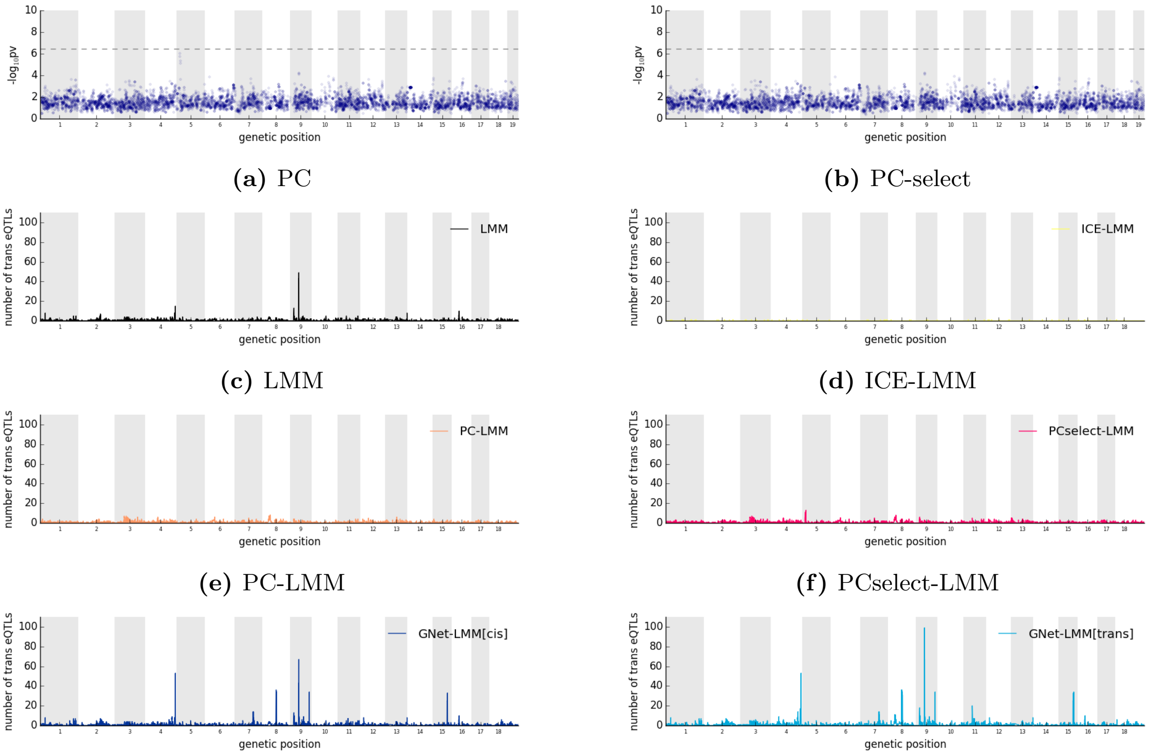

(a) PC

(b) PC-select

(c) LMM

(d) ICE-LMM

(e) PC-LMM

(f) PCselect-LMM

(g) GNet-LMM[cis]

(h) GNet-LMM[trans]

Figure S8: **The latent factors learnt by PC-based methods are associated with putative *trans* hotspots.** Shown are manhattan plots of the minimum p-value for the 20 principle components used in PC LMM (**a**) or PC-select LMM on the mouse dataset (**b**, after removal of PCs with strong genetic association, Methods). (**c- h**) the number of *trans*-genes in association with genome-wide variants, considering the threshold $\alpha < 10^{-4}$, for alternative models. Several of the putative *trans* hotspots recovered by a standard LMM and GNET-LMM models are co-located with suggestive associations with the principle components learnt by PC LMM and PC-select LMM. Consequently, conditioning on these principle components results in loss of power (**e,f**).

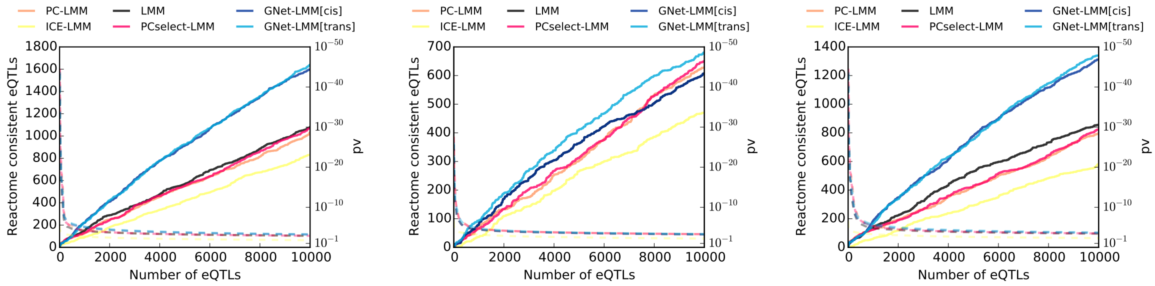

(a) Cis **(b)** No Cis **(c)** All

Figure S9: **Reactome enrichment analysis of eQTLs identified on the mouse dataset.** Enrichment analysis as in main Figure 3, however stratifying genome-wide tests for the presence or absence of a *cis* anchor. Solid lines show the enrichment, dashed lines the corresponding p-value threshold. (**a**) tests with a *cis*-anchor (pv$<10^{-6}$) (**b**), tests without a cis anchor (pv$>10^{-6}$) and (**c**), all tests. Tests with a *cis* anchor gene enable the detection of V-structures using GNetLMM[cis], resulting in equal performance of GNetLMM[cis] and GNetLMM[trans]. In contrast, for tests without *cis* eQTLs, GNETLmm[cis] reverts to a standard LMM whereas GNETLmm[trans] still achieves a greater enrichment than other methods. Overall, there appears to be a stronger enrichment of pathway-consistent associations among tests that do have *cis* anchors, which is consistent with the idea that genuine *trans* associations tend to be *cis* mediated.

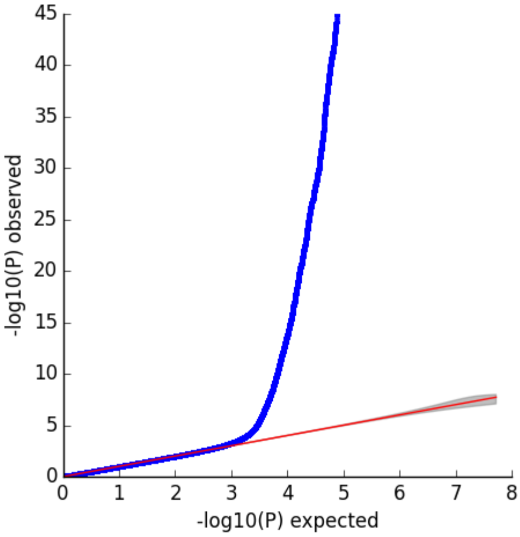

(a) not permuted, without exogenous genes

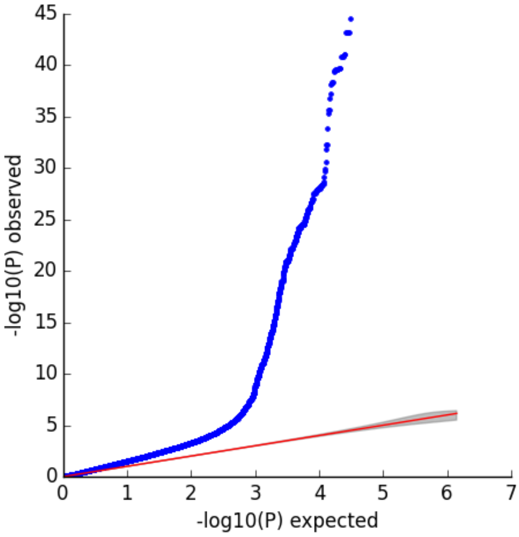

(b) not permuted, with exogenous genes

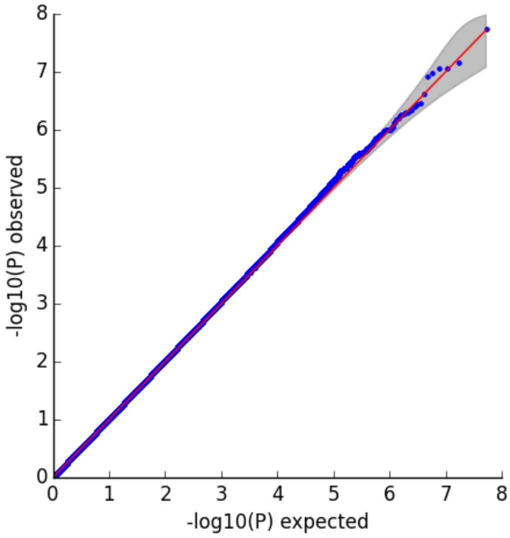

(c) permuted, without exogenous genes

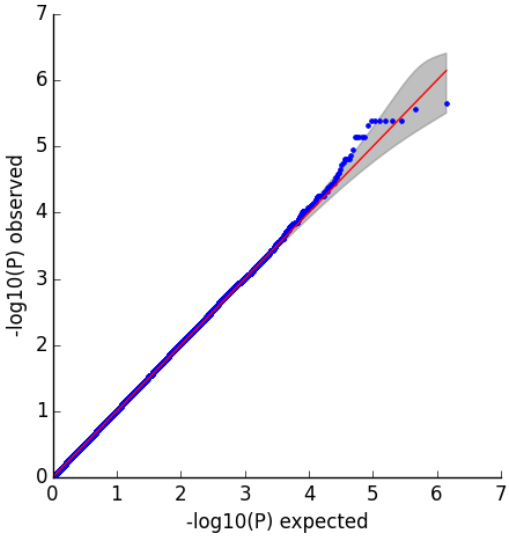

(d) permuted, with exogenous genes

Figure S10: **Quantile-quantile plots on the mouse dataset.** Shown are quantile-quantile plots of association results from GNetLMM[cis] on real data (top) and on permuted data (bottom), permuting the SNPs for association testing while retaining the background model. **Left:** p-values from tests without exogenous genes (no V-structures identified), **right:** p-values from tests with exogenous variables. On permuted data, the background model was retained, including the random effect covariance that accounts for exogenous genes as identified in the V-structure search of GNetLMM. We used different permutation on the gene level, but kept the permutation fixed across the markers when testing for the same gene. The results show that the association p-values obtained from GNetLMM are well calibrated, both for tests with and without exogenous genes.

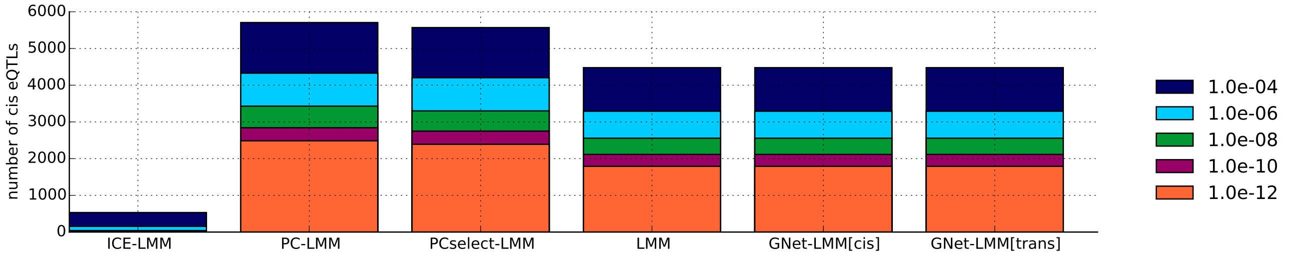

Figure S11: **Number of *cis* associations retrieved by alternative methods for varying p-value thresholds on the mouse dataset.** Reported are the number of *cis*-associations (± 2 mb of the TSS) for different thresholds. For *cis* associations, GNet-LMM[cis/trans] is identical to a standard LMM as no V-structures are identified. In contrast, PC-LMM and PCselect-LMM help to boost power for *cis*-eQTL mapping, which is consistent with previously reported results.

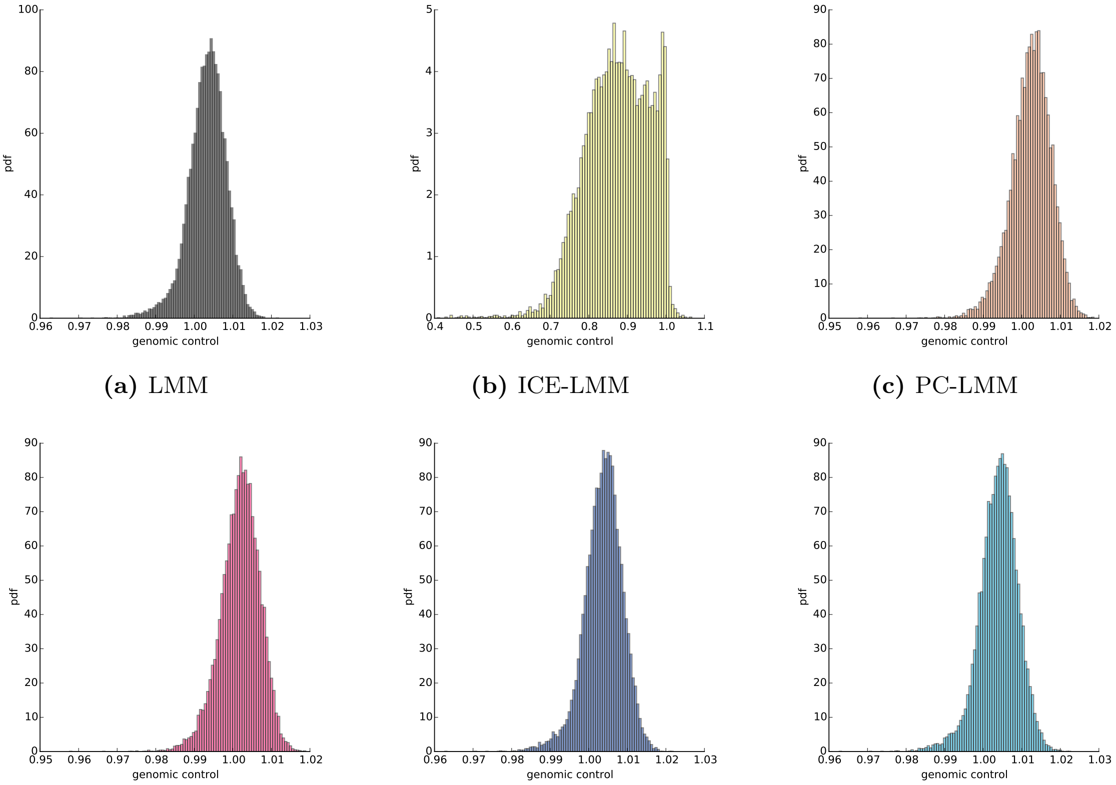

(a) LMM **(b)** ICE-LMM **(c)** PC-LMM

(d) PCselect-LMM **(e)** GNet-LMM[cis] **(f)** GNet-LMM[trans]

Figure S12: **Calibration of alternative methods on the Cardiogenics dataset.** Shown are histograms of genomic control values estimated for *trans* test (distance to the TSS $>$ 2mb) for each individual gene. All methods except for LMM-ICE were well calibrated.

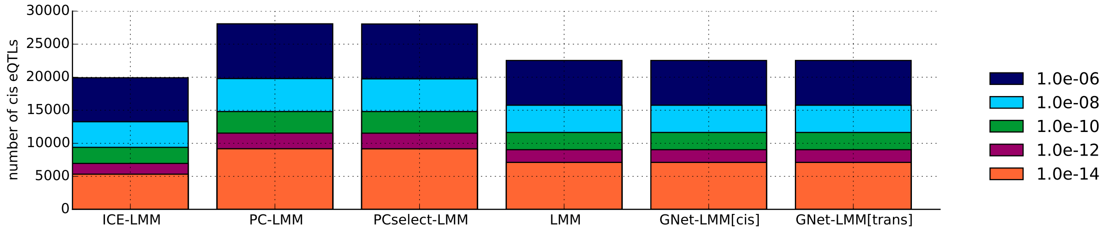

Figure S13: **Number of retrieved *cis* associations on the Cardiogenics dataset.** Shown is the number of *cis*-hits found for varying cutoff values. Across all thresholds, factor model based approach find more hits than standard approaches. As expected, GNet-LMM performs equivalently to the standard LMM.

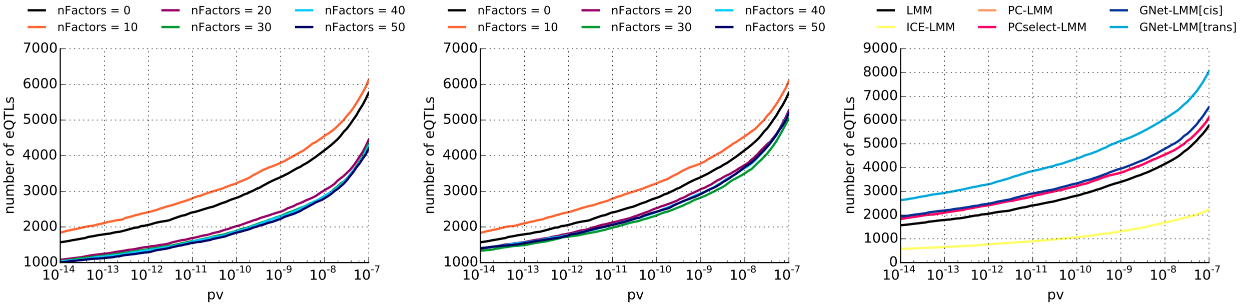

(a) PC-LMM **(b)** PCselect-LMM **(c)** ALL

Figure S14: **Number of *trans* associations associations retrieved by alternative methods on the Cardiogenics dataset.** Shown is the number of *trans*-associations as a function of the p-value threshold for PC-LMM (**a**) and PCselect-LMM (**b**), considering alternative numbers of principle components to estimate hidden factors. **c)** shows the number of associations retrieved by all methods (using 10 principle components of PC-LMM and PCselect-LMM). For all further analyses, the setting of 10 principal components was considered for PC-based methods.

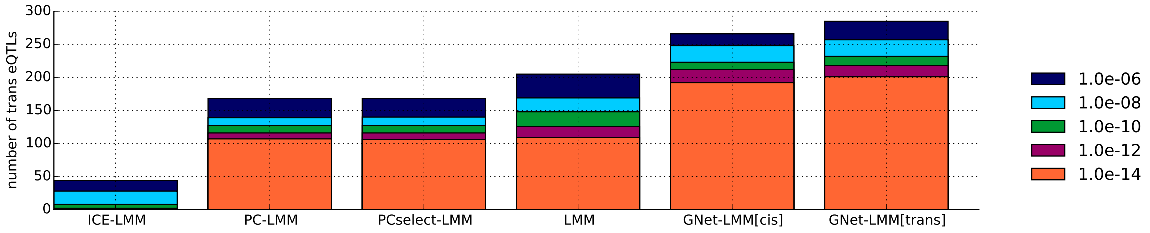

Figure S15: **Number of *trans*-genes identified downstream of the marker rs6581889 in the LYZ region for varying cutoff values on the Cardiogenics dataset.** Shown is the number of genes in association with rs6581889 for alternative methods. Across all pv-threshold, methods based on principle components (PC-LMM, PCselect-LMM) identify fewer *trans* eQTls than a standard LMM. In contrast, GNet-LMM[cis/trans] outperform these methods and increase power.

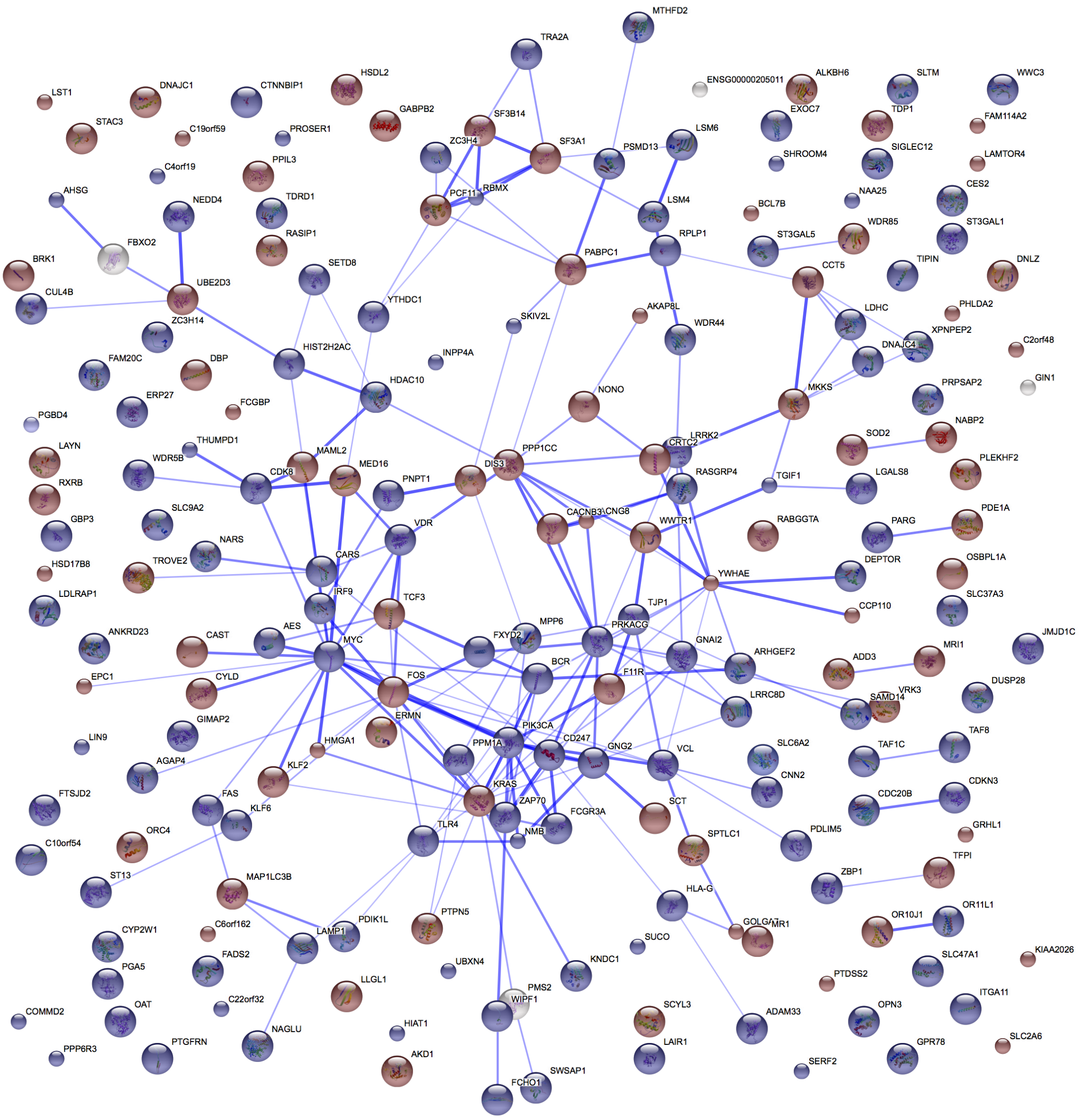

Figure S16: **String Network for genes that are associated with the LYZ region ($pv < 0.01$, Bonferroni adjusted) on the cardiogenics dataset.** Genes marked in blue were identified both by Gnet-LMM[cis] and LMM, genes marked in red were exclusively identified by GNet-LMM[cis]. There were no genes that are identified by the LMM and missed by GNetLMM[cis]. The light grey genes could not be identified and are either found by LMM and Gnet-LMM[cis], or by only the latter one.

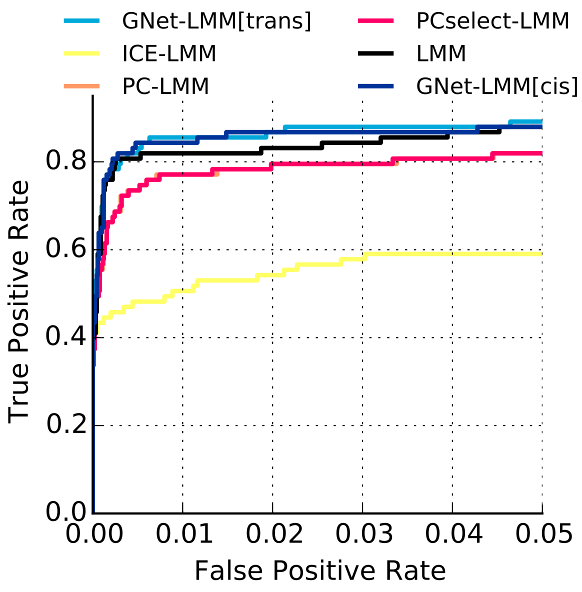

Figure S17: **Validation of genes that are associated with the LYZ region on the Cardiogenics dataset using an independent Monocyte eQTL study.** Shown is the receiver operating characteristics (ROC) for eQTLs discovered in the Cardiogenics dataset using different methods, when using eQTL genes discovered in an independent Monocyte eQTL dataset (Fairfax et al., 2014) as ground truth set (standard linear mixed model; P < 0.01, Bonferroni adjusted across tests). The ROC curve is truncated at an FPR of 5%. The replication rate was best for GNet-LMM, whereas other methods resulted in a lower replication rate than obtained using a standard linear model.

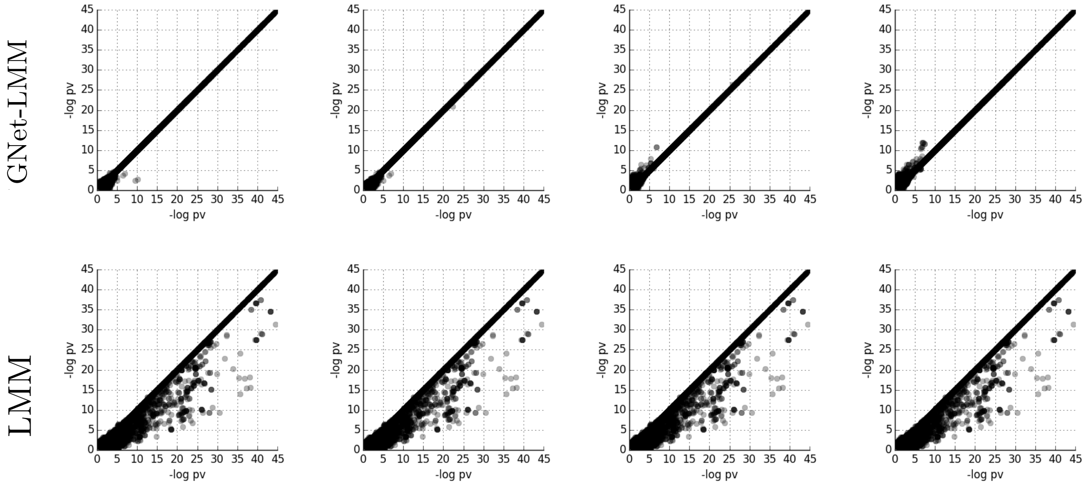

(a) $\alpha_{cis} = 10^{-4}$ **(b)** $\alpha_{cis} = 10^{-5}$ **(c)** $\alpha_{cis} = 10^{-7}$ **(d)** $\alpha_{cis} = 10^{-8}$

Figure S18: **Consistency of GNet-LMM p-values for varying *cis*-thresholds on the mouse dataset. top panel:** Paired p-values (log-transformed), comparing between GNet-LMM[cis] with alternative parameter settings (x-axis) and the default parameter values ($\alpha_{cis} = 10^{-6}$) as used for the results in the main manuscript (y-axis). **bottom panel:** Analogous comparison of paired p-values between GNet-LMM[cis] results for alternative parameter settings (x-axis) with p-values from a standard LMM. Overall, the differences between parameter settings of GNetLMM[cis] are small compared to differences between methods (bottom row), suggesting that GNetLMM is robust w.r.t. threshold values.

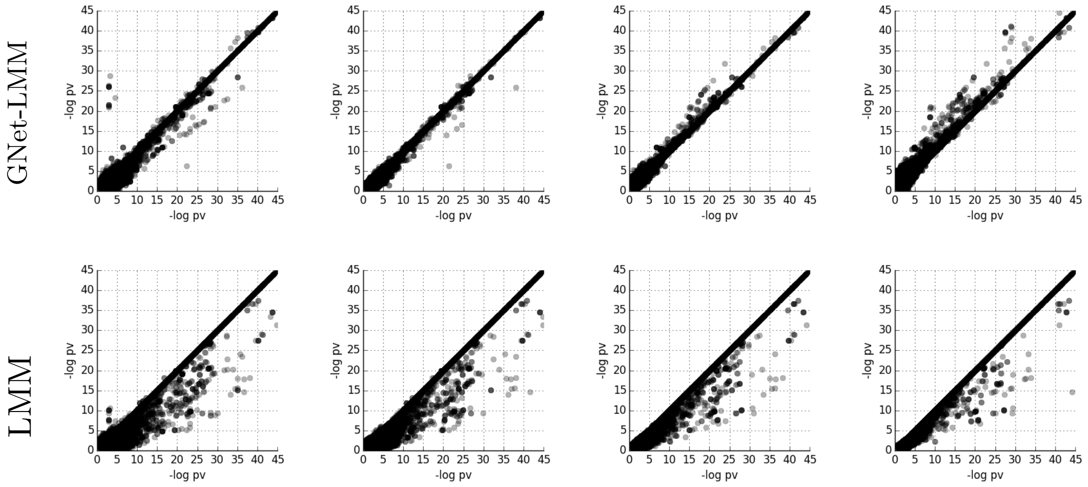

(a) $\alpha_{ind} = 0.01$ **(b)** $\alpha_{ind} = 0.05$ **(c)** $\alpha_{ind} = 0.2$ **(d)** $\alpha_{ind} = 0.5$

Figure S19: **Consistency of GNet-LMM p-values for varying adjusted *ind*-thresholds on the mouse dataset.** Paired p-values (log-transformed), comparing between GNet-LMM[cis] with alternative parameter settings (x-axis) and the default parameter values ($\alpha_{ind} = 0.1$) as used for the results in the main manuscript (y-axis). **bottom panel:** Analogous comparison of paired p-values between GNet-LMM[cis] results for alternative parameter settings (x-axis) with p-values from a standard LMM. Overall, the differences between parameter settings of GNetLMM[cis] are small compared to differences between methods (bottom row), suggesting that GNetLMM is overall robust w.r.t. threshold values.

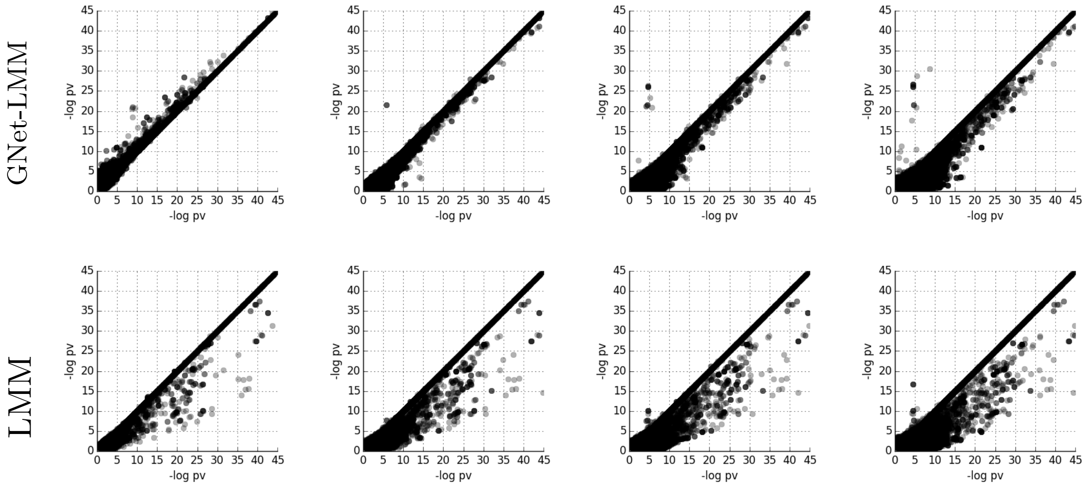

(a) $\alpha_{assoc} = 0.005$ **(b)** $\alpha_{assoc} = 0.05$ **(c)** $\alpha_{assoc} = 0.1$ **(d)** $\alpha_{assoc} = 0.2$

Figure S20: **Consistency of GNet-LMM p-values for varying adjusted *assoc*-thresholds on the mouse dataset. top panel:** Paired p-values (log-transformed), comparing between GNet-LMM[cis] with alternative parameter settings (x-axis) and the default parameter values ($\alpha_{assoc} = 0.01$) as used for the results in the main manuscript (y-axis). **bottom panel:** Analogous comparison of paired p-values between GNet-LMM[cis] results for alternative parameter settings (x-axis) with p-values from a standard LMM. Overall, the differences between parameter settings of GNetLMM[cis] (top row) are small compared to differences between methods (bottom row), suggesting that GNetLMM is overall robust w.r.t. threshold values.

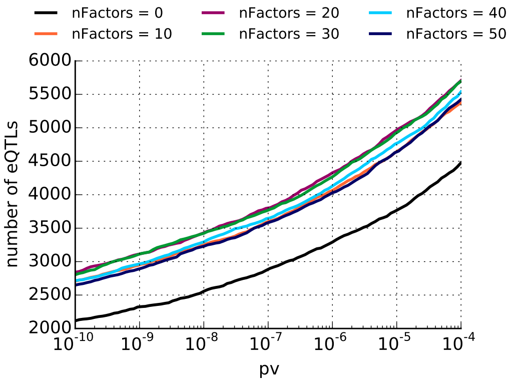

(a) PC-LMM (cis)

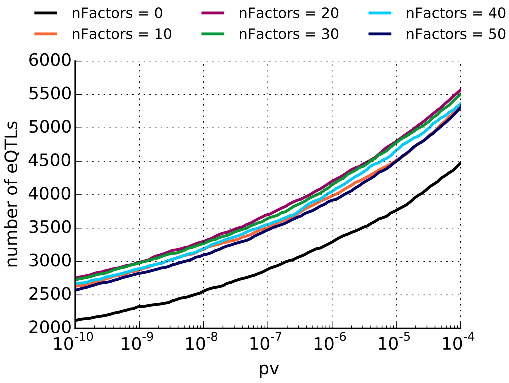

(b) PC-LMMselect (cis)

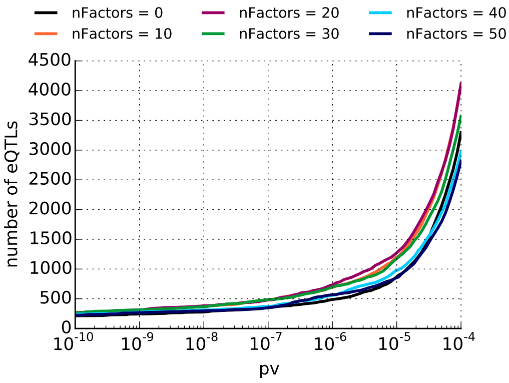

(c) PC-LMM (trans)

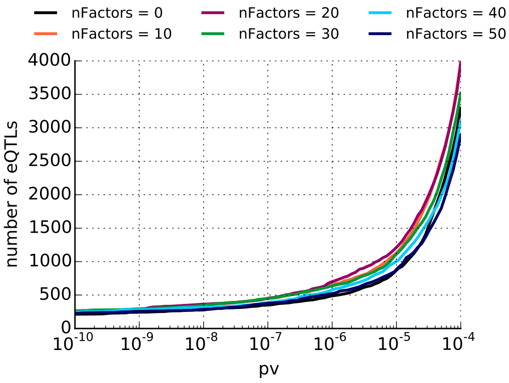

(d) PCselect-LMM (trans)

Figure S21: **Selection of the number of principle components for PC-based methods on the mouse dataset.** Shown are the number of significant *cis* (top panel) and *trans*-associations (bottom panel) as a function of the p-values thresholds and when considering alternative numbers of principle components in the model. Left: PC-LMM, Right: PCselect-LMM. For the main results reported, we considered the setting of 20 principle components for both models.

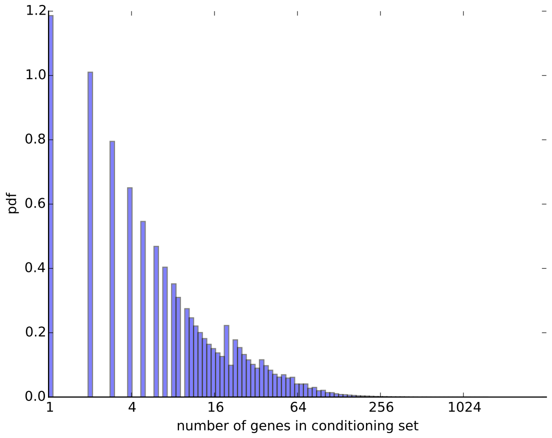

Figure S22: **Distribution of the number of genes in the conditioning set for the Cardiogenic dataset.** Shown is the distribution of the number of genes per unique conditioning set on the Cardiogenics dataset. Most conditioning sets contain only a handful of genes, making the inference amenable for low-rank tricks.
